# Integrative analysis revealed the molecular mechanism underlying RBM10-mediated splicing regulation

**DOI:** 10.1002/emmm.201302663

**Published:** 2013-08-22

**Authors:** Yongbo Wang, Andreas Gogol-Döring, Hao Hu, Sebastian Fröhler, Yunxia Ma, Marvin Jens, Jonas Maaskola, Yasuhiro Murakawa, Claudia Quedenau, Markus Landthaler, Vera Kalscheuer, Dagmar Wieczorek, Yang Wang, Yuhui Hu, Wei Chen

**Affiliations:** 1 Laboratory for Novel Sequencing Technology, Functional and Medical Genomics, Berlin Institute for Medical Systems Biology, Max-Delbrueck-Center for Molecular MedicineBerlin, Germany; 2 Max-Planck-Institute for Molecular GeneticsBerlin, Germany; 3 Department of Clinical Laboratory, Taiyuan Central HospitalTaiyuan, Shanxi, P. R. China; 4 Laboratory for Systems Biology of Gene Regulatory Elements, Berlin Institute for Medical Systems Biology, Max-Delbrueck-Center for Molecular MedicineBerlin, Germany; 5 Laboratory for RNA Biology and Posttranscriptional Regulation, Berlin Institute for Medical Systems Biology, Max-Delbrueck-Center for Molecular MedicineBerlin, Germany; 6 Institut für Humangenetik, Universitätsklinikum EssenEssen, Germany; 7 Department of Pharmacology, Lineberger Comprehensive Cancer Center, University of North CarolinaChapel Hill, North Carolina, USA

**Keywords:** RBM10, alternative splicing, mechanistic model

## Abstract

*RBM10* encodes an RNA binding protein. Mutations in *RBM10* are known to cause multiple congenital anomaly syndrome in male humans, the TARP syndrome. However, the molecular function of RBM10 is unknown. Here we used PAR-CLIP to identify thousands of binding sites of RBM10 and observed significant RBM10–RNA interactions in the vicinity of splice sites. Computational analyses of binding sites as well as loss-of-function and gain-of-function experiments provided evidence for the function of RBM10 in regulating exon skipping and suggested an underlying mechanistic model, which could be subsequently validated by minigene experiments. Furthermore, we demonstrated the splicing defects in a patient carrying an *RBM10* mutation, which could be explained by disrupted function of RBM10 in splicing regulation. Overall, our study established RBM10 as an important regulator of alternative splicing, presented a mechanistic model for RBM10-mediated splicing regulation and provided a molecular link to understanding a human congenital disorder.

## INTRODUCTION

Alternative splicing (AS) is considered as the major mechanism that contributes to the increased proteomic diversity in multicellular eukaryotes (Blencowe, 2006; Maniatis & Tasic, 2002; Nilsen & Graveley, 2010). Through AS, one pre-mRNA could produce multiple mRNA isoforms that might be under different post-transcriptional regulation and/or encode proteins with different functions. Recent transcriptome analysis by massive parallel RNA sequencing (RNA-seq) indicated that more than 90% human genes underwent AS (Pan et al, 2008; Wang et al, 2008). Very often, the pattern of AS was tissue and developmental stage specific, thought to be under precise regulation modulated by cooperative interplays between *trans*-acting RNA binding proteins (RBPs) and *cis*-regulatory elements in nascent transcripts (Barash et al, 2010; Black, 2003; Chen & Manley, 2009; Witten & Ule, 2011). Mutations in splicing regulators (Padgett, 2012; Yoshida et al, 2011) and abnormal splicing of RNA targets have been associated with many human diseases (Cooper et al, 2009; Garcia-Blanco et al, 2004; Wang & Cooper, 2007). Nevertheless, the exact molecular mechanisms controlling the AS process in physiological and pathological conditions are not well-understood to date. Several splicing regulating RBPs have recently been found to modulate hundreds even thousands of functional targets (Lebedeva et al, 2011; Licatalosi et al, 2012; Mukherjee et al, 2011; Ule et al, 2006; Wang et al, 2012; Xue et al, 2009; Yeo et al, 2009). Therefore, elucidating the regulatory roles of splicing related RBPs requires comprehensive identification of the RBP–RNA interactions and global quantification of the splicing outcomes induced by RBPs.

*RBM10* encodes a 930 amino acid protein containing two RNA recognition motifs (RRM), two zinc fingers and one G patch motif. These motifs were often found in RNA-binding proteins involved in pre-mRNA splicing, such as heterogeneous nuclear ribonucleoproteins (hnRNPs) and protein components of small nuclear ribonucleoproteins (snRNPs; Glisovic et al, 2008; Keene, 2007). Through mass spectrometric analysis, RBM10 has been reported to associate with purified splicing complex (Rappsilber et al, 2002), and was further identified as a component of U2 snRNPs (Makarov et al, 2011), spliceosomal A (or prespliceosomal) (Agafonov et al, 2011; Behzadnia et al, 2007) and B complexes (Agafonov et al, 2011; Bessonov et al, 2008). Most recently, based on yeast two hybridization method, a study on interactions between more than 200 proteins previously known to be present in spliceosome could demonstrate the physical interaction between RBM10 and multiple spliceosomal components (Hegele et al, 2012). Moreover, its closest paralogue RBM5, a putative tumour suppressor of lung and other cancers (Sutherland et al, 2005), has been shown to regulate AS of apoptosis related genes, *Fas* receptor and c-*FLIP*, resulting in isoforms with antagonistic functions in controlling programmed cell death (Bonnal et al, 2008). Although all these observations would suggest the potential role of RBM10 in pre-mRNA splicing regulation, it remains unclear whether and how RBM10 could regulate splicing. Nonsense and frame shift mutations in *RBM10* have been identified to be causative for TARP syndrome (Talipes equinovarus, atrial septal defect, Robin sequence and persistent left superior vena cava, MIM #311900), an X-linked inherited disorder leading to multiple organ malformation in affected males (Gripp et al, 2011; Johnston et al, 2010). More recently, multiple truncating and missense somatic mutations were detected in lung adenocarcinomas (Imielinski et al, 2012). These findings implicated the important role of RBM10, but whether its potential function in splicing regulation is involved in these different pathological contexts has not been explored.

In this study, we explored the AS regulated by RBM10. Here, we combined photoactivatable-ribonucleoside-enhanced crosslinking and immunoprecipitation (PAR-CLIP) with massive parallel sequencing to identify RNA binding sites for RBM10 in human embryonic kidney (HEK) 293 cells, which turned out to be significantly enriched in the vicinity of both 5′ and 3′ splice sites. Using RNA-seq, we identified 304 and 244 significant exon splicing changes following RBM10 depletion or overexpression (OE) in HEK293 cells, respectively. Among these changes, more than 74% were RBM10 enhanced exon skipping events and they were correlated with strong RBM10 binding near 5′ and 3′ splicing sites of both upstream and downstream introns. Furthermore, in a patient suffering from TARP syndrome, we identified an *in-frame* deletion in *RBM10* and demonstrated that the splicing defects in the lymphoblastoid cells derived from the patient were largely due to the loss of nuclear function of RBM10. Overall, our data provides direct experimental evidence supporting the role of RBM10 in splicing regulation. Our transcriptome-wide analysis of binding pattern and RBM10 splicing profile allows the illustration of the molecular mechanism underlying RBM10 regulated AS.

## RESULTS

### Transcriptome-wide binding sites of RBM10 in HEK293 cells

To identify *in vivo* binding sites of RBM10, we performed PAR-CLIP sequencing (Hafner et al, 2010; Lebedeva et al, 2011) in HEK293 cells that expressed epitope (FLAG/HA)-tagged RBM10 (Materials and Methods Section). 4-Thiouridine (4SU) labelled and crosslinked cells were immunoprecipitated with monoclonal anti-FLAG antibody. The bound RNAs was then partially digested and radioactively labelled. Protein–RNA complexes were resolved on a denaturing gel. The band corresponding to RBM10–RNA complexes was excised (Supporting Information Fig S1A). The RNA was recovered, converted into cDNA and sequenced on an Illumina platform. In total, we performed two biological replicate experiments. The sequencing reads were processed and clustered as described in Materials and Methods Section.

A total of 20.6 million sequencing reads could be mapped to the human genome with at most one mismatch (Supporting Information Table S1). Compared with all other mutations in the mappable sequence reads, T to C transitions were significantly enriched (Supporting Information Fig S1B), manifesting efficient crosslinking of 4SU labelled RNA (Hafner et al, 2010). We identified 240,712 and 218,281 RBM10 sequence clusters (putative binding sites) in the two replicates, respectively (Supporting Information Table S1, Fig S1C and D for the length distribution of binding clusters as well as the number of PAR-CLIP reads within each cluster). Of these, 87,957 sequence clusters had their preferred crosslinking sites, *i.e*. the position with the highest number of T to C transitions within a site, to be within the binding site identified in the other replicate. We defined these clusters as consensus binding clusters. Comparison of the binding scores of these consensus binding clusters between the two replicates revealed a high correlation (*R*^2^ = 0.619) (Supporting Information Fig S1E).

### RBM10 binding in the vicinity of intronic splicing sites

**Figure 1:**
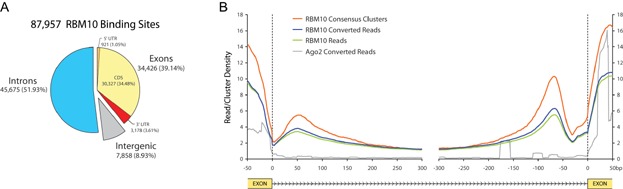
RBM10 RNA binding sites identified by PAR-CLIP The genomic distribution of 87,957 RBM10 consensus binding clusters.Distribution of RBM10 consensus binding clusters (red line), PAR-CLIP reads (green line) and PAR-CLIP reads containing at least one T to C change (converted reads, blue line) along exon–intron and intron–exon boundaries. The density of RBM10 converted PAR-CLIP reads in the vicinity of intronic splice sites were significantly higher than that of Ago2 converted PAR-CLIP reads (grey line).

Ninety-one percent of the consensus binding sites could be assigned to 6396 RBM10 target genes. According to Refseq annotation, 39 and 52% of them fell into exonic and intronic regions, whereas 9% mapped to intergenic regions, which might harbour previously unannotated transcripts (Fig 1A). Given the possible involvement of RBM10 in splicing process, we examined the distribution of binding sites relative to splice sites. Intriguingly, we found that they were significantly enriched in exons and in the vicinity of both 5′ and 3′ splice sites of the introns (Fig 1B). Notably, the binding sites were more enriched at the vicinity of (∼70 nt upstream) of 3′ splice site than at 5′ splice site. Interestingly, we also observed the specific binding of RBM10 at U2 snRNA, which is known to pair with 3′ branch site (Supporting Information). Together, these binding patterns were consistent with the previous findings of RBM10 in pre-spliceosomal A and B complex (Agafonov et al, 2011; Behzadnia et al, 2007; Bessonov et al, 2008) and indicated that RBM10 very likely involves in splice site recognition and/or pairing, as well as further intron removal processes via coordinated interactions with snRNPs and the pre-mRNA substrates.

### Alternative splicing regulated by RBM10

The RNA binding patterns presented above suggested that RBM10 might function as a splicing regulator. To test this possibility, we performed RNA-seq and quantified changes in gene expression as well as AS in HEK293 cells upon RBM10 knockdown (KD) or overexpression (OE) respectively (Materials and Methods Section). The efficiency of KD and OE were assessed both at mRNA level by qPCR and at protein level by Western blot (Supporting Information Fig S2A and B). In total, we performed two and four biological replicate experiments for OE and KD, respectively. Sixty-one to 185 millions 100 nt sequencing reads were generated for each sample, of which 92–96% could be mapped to the genome reference (UCSC genome browser hg19) or a reference set of exon–exon junction sequences (see Materials and Methods Section and Supporting Information Table S2).

The gene expression level was estimated based on RPKM value (reads per kilobase of exon per million mapped sequence reads, (Mortazavi et al, 2008), Materials and Methods Section). At false discovery rate (fdr) <0.05, 171 and 105 genes were found to be significantly upregulated and downregulated by at least 1.5-fold upon RBM10 KD (Supporting Information Fig S2C and Table S3), whereas 19 and 49 genes were upregulated and downregulated to the same level (fdr < 0.05, fold change ≥1.5) in response to RBM10 OE, respectively (Supporting Information Fig S2D and Table S3). Overall, the expression changes induced by KD and OE were not inversely correlated (Supporting Information Fig S2E).

**Figure 2:**
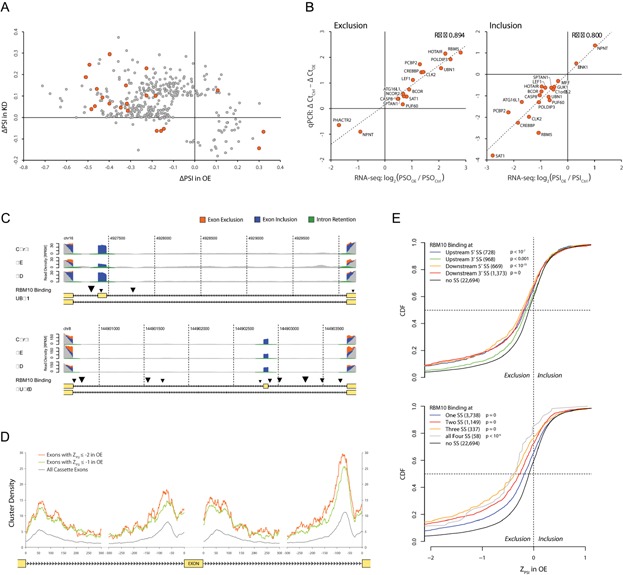
Exon splicing changes induced by RBM10 perturbation and their association with RBM10 binding profile Exon splicing changes (ΔPSI, percentage splicing in) induced by RBM10 OE (*X* axis) were plotted against those induced by RBM10 KD (*Y* axis). A total of 412 cassette exons were found to be differentially spliced after RBM10 OE or KD (FDR ≤ 5%, |ΔPSI| ≥ 0.1).The splicing changes (left panel, ΔPSO, percentage splicing out; right panel, ΔPSI) of 21 exons (orange dots in A) quantified by RNA-seq (*X* axis) are highly correlated with those measure by qPCR (*Y* axis).Representative examples of RBM10 dependent exon skipping events. The density of RNA-seq reads obtained in control, RBM10 OE and KD experiments together with RBM10 binding sites were shown for gene *UBN1* and *PUF60*. Reads supporting the inclusion or exclusion of the cassette exon, or spanning exon–intron junctions were shown in blue, orange or green, respectively. The remaining reads were shown in grey. The size of the triangle marking RBM10 binding sites reflected the number of PAR-CLIP reads.The density of RBM10 binding clusters close to 5′ and 3′ splicing sites of introns flanking all cassette exons (black) is significantly lower than those flanking the exons more excluded after RBM10 OE (orange, *Z*_PSI_ ≤−2 and green, *Z*_PSI_ ≤−1).Cumulative distribution functions of splicing changes upon RBM10 OE for different groups of cassette exons with RBM10 binding close to none or one of the four splicing sites (upper panel), or to different number of the four splicing sites (lower panel). The numbers of exons within different groups were printed in parenthesis.

We then sought to characterize the splicing changes induced by RBM10 OE/KD. Based on RNA-seq data, we defined the inclusion ratio (PSI: percentage splicing in) of each exon in Refseq transcripts as the number of reads exclusively supporting inclusion divided by total number of reads supporting inclusion and exclusion of the specific exon (Supporting Information Fig S2F; Polymenidou et al, 2011; Wang et al, 2008). We then compared the inclusion ratio between KD and control, OE and control, respectively. The changes were transformed into *Z*-value (Supporting Information Fig S2H) and the results from replicate experiments were combined to evaluate statistical significance using the rank product method (Materials and Methods Section). At a stringent cutoff (fdr < 0.05, |ΔPSI| ≥ 10%), we identified 256 induced cassette exon inclusion and 48 exclusion events upon RBM10 KD (Fig 2A and Supporting Information Table S4). In comparison, 27 exon inclusion and 217 exclusion events were observed upon RBM10 OE (Fig 2A and Supporting Information Table S4).

We then selected 21 candidate transcripts for which we had detected splicing changes with different *Z* values for validation by qPCR using junction specific primers (Fig 2A and Supporting Information Fig S3). The abundance of transcript isoforms including or excluding the cassette exons was normalized based on that of constitutive exons. We could validate splicing changes in all the 21 cases (Supporting Information Fig S3). The splicing changes detected by qPCR were quantitatively correlated with that determined by RNA-seq (Fig 2B). Comparison of splicing changes induced by OE and that by KD revealed a clear inverse correlation (Fig 2A). The majority (74%) of the splicing changes observed upon RBM10 OE and KD are RBM10-enhanced exon exclusion events (Fig 2A), indicating that RBM10 primarily mediated the skipping of cassette exons.

### An RNA splicing map integrating RBM10 binding profile and induced splicing changes

We then took a close look at functional annotations of the genes that changed the expression level and/or the splicing pattern as a result of RBM10 perturbation. Interestingly, 14 RNA-binding proteins and five known splicing regulators were found with significant expression changes (Supporting Information Table S3), and even more genes with splicing changes (22 and eight) were found to be involved in RNA-binding or splicing regulation (Supporting Information Table S4). Therefore, the overall splicing changes described above represented not only the direct RBM10-targeted splice events, but also the secondary effects resulting from the expression and/or splicing changes in those splicing regulators. In order to understand the mechanisms for AS directly under RBM10 regulation, we correlated the RBM10 RNA binding pattern with the splicing changes upon RBM10 OE and KD. Among the RBM10-enhanced exon skipping events, we often observed RBM10 binding(s) close to 5′ and/or 3′ splicing sites at upstream and/or downstream introns. Two representative examples were depicted in Fig 2C. To examine general mechanism for RBM10 enhanced exon skipping, we integrated the PAR-CLIP data and splicing profiles into an RNA splicing map. As shown in Fig 2D, the map revealed increased density of RBM10 binding clusters close to the splice sites of both introns flanking the skipped cassette exons, with the most prominent enrichment at 3′ splice site of downstream intron.

The RNA splicing map of RBM10 suggested that RBM10 binding close to the splice sites of neighbouring introns are enriched for skipped exons. To assess whether such binding pattern could predict exon exclusion events, we searched our PAR-CLIP data for non-constitutive exons with RBM10 binding close (*i.e*. up to 150 nt) to the splice sites of adjacent introns. In total, 5262 such exons were found. Among these exons, 147 showed significant splicing changes (fdr < 0.05, |ΔPSI| ≥ 10%) upon RBM10 OE and/or KD, accounting for 30.8% (147/412) of all the exons with significant splicing changes upon RBM10 perturbation. As shown in Fig 2E, the exons with RBM10 binding close to one of the four splice sites were more likely excluded upon RBM10 OE, and those with binding close to 3′ splice sites of upstream introns exhibiting the weakest skipping propensity. Intriguingly, exons with binding close to more of the four splice sites showed progressively stronger skipping tendency upon RBM10 OE (Fig 2E). Similarly, exons with binding close to the same four splicing junctions showed progressively stronger inclusion tendency upon RBM10 KD (Supporting Information Fig S4).

### Mechanistic study of RBM10 enhanced exon skipping using minigenes

**Figure 3:**
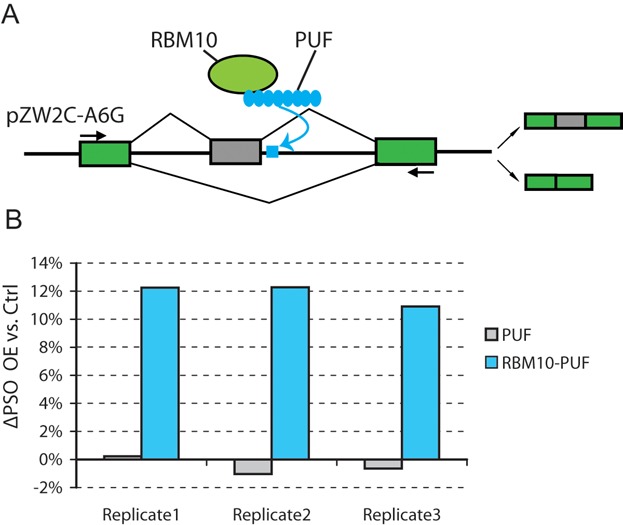
Validation of RBM10-enhanced exon exclusion with minigene experiments Schematic representation of the minigene experiment, in which RBM10 was tethered to an intronic region 18-nt downstream of the middle cassette exon via fusing to the PUF domain that recognizes its cognate sequence (blue rectangle).Splicing changes of the cassette exons upon the OE of RBM10-PUF fusion protein or PUF alone, detected in three biological replicates. RBM10 tethered in the vicinity of intronic splice site could significantly promote exon skipping (two-tailed paired *t*-test *p*-value: 0.001832).

We demonstrated that RBM10 binding near splice sites of flanking introns would enhance the skipping of cassette exons. In order to test the direct effect of the RBM10 binding on pre-mRNA splicing, we fused RBM10 with a modified pumilio domain, PUF3-2, which specifically recognizes an eight nucleotide sequence ‘UGUAUGUA’ with high affinity (Fig 3A; Wang et al, 2009). By co-transfecting the RBM10-PUF fusion protein with the splicing reporter pZW2C-A6G that contains PUF cognate sequence, we could tether RBM10 to an intronic region 18-nt downstream of the middle exon (Fig 3A; Wang et al, 2009, 2013). Whereas the expression of PUF domain alone hardly induced any splicing changes, the expression of RBM10-PUF fusion protein exhibited strong exon skipping effects (Fig 3B and Supporting Information Fig S5), providing unequivocal support to our hypothesis that RBM10 intronic binding in the vicinity of splicing sites would facilitate the skipping of cassette exons.

### Mechanistic model underlying RBM10 regulated alternative splicing

**Figure 4:**
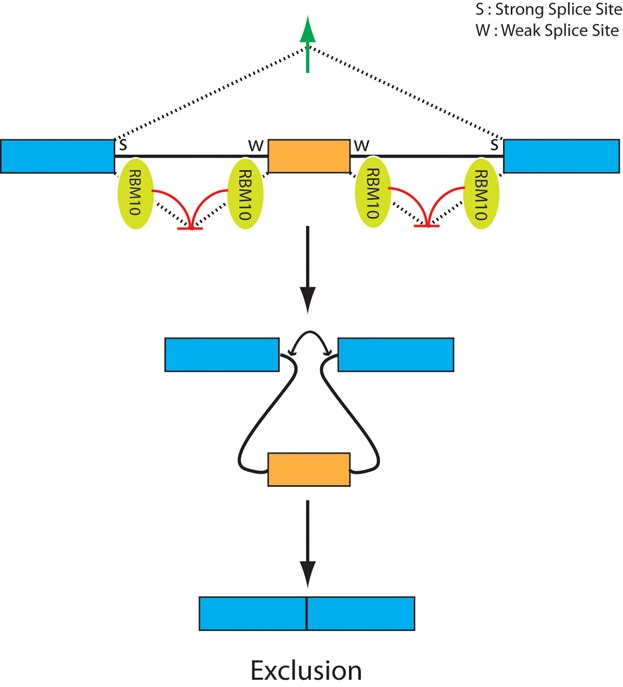
Mechanistic model for RBM10-mediated splicing regulation RBM10 binding in the vicinity of splicing sites might repress the splicing of flanking introns and delay the splicing choice, thereby facilitate the skipping of cassette exons flanked with relatively weaker splice sites.

**Figure 5:**
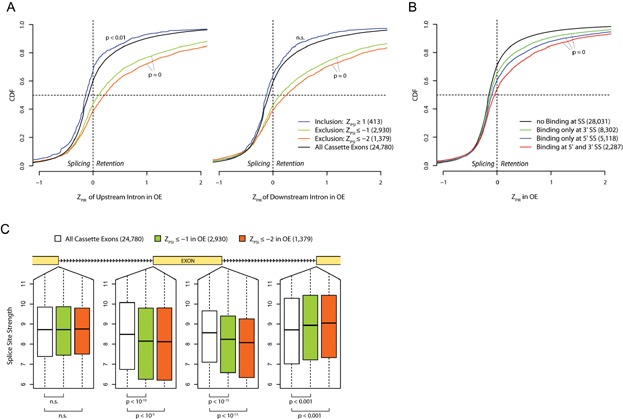
Correlation between RBM10 binding, exon splicing changes, intron splicing changes and splicing site strengths Cumulative distribution functions (CDF) of splicing change of introns (*Z*_PIR_, percentage intron retention) upstream (left) or downstream (right) to the cassette exons that were differentially spliced upon RBM10 OE. Exon exclusion upon RBM10 OE (orange: *Z*_PSI_ ≤−2; green: *Z*_PSI_ ≤−1, PSI: percentage splicing in) is associated with higher retention of both flanking introns than background (black) and exon inclusion (blue: *Z*_PSI_ ≥1). The numbers of exons were indicated in parenthesis.Cumulative distribution functions of *Z*_PIR_ for different groups of introns with or without RBM10 binding at the 5′ and/or 3′ splice sites. The numbers of introns within different groups were printed in parenthesis.Box plots of the strengths of splicing splice sites at upstream and downstream introns flanking all cassette exons (white) or those with higher exclusion after upon RBM10 OE (orange: *Z*_PSI_ ≤−2; green: *Z*_PSI_ ≤−1).

RBM10 binding close to splice sites might interfere with splicing sites recognition and/or splice sites pairing. Our observations strongly supported the model that RBM10 binding in the vicinity of splicing sites might repress the splicing of introns and delay the splicing choice; thereby facilitate the skipping of cassette exons flanked with relatively weaker splicing sites (Fig 4). Indeed, we found several lines of evidences supporting such a working model. First, a clear positive correlation between exon skipping and the retention of flanking introns could be observed based on RNA-seq results (Fig 5A). Second, the intron retention appeared to be also enhanced by RBM10 binding in the vicinity of its splice sites, especially the binding near 5′ splice sites (Fig 5B). Third, we proposed that the exon skipping was largely due to the weaker flanking splice sites. Once the splicing of flanking intron was repressed, the use of stronger distal splicing sites would be enhanced. As shown in Fig 5C, the strength of splicing sites distal to the cassette exons was generally stronger than that of those immediately flanking the exons. Finally, RBM10 binding close to downstream 3′ splice sites might also facilitate their paring with upstream 5′ splicing sites at later steps of splicing process, an effect proposed previously by Bonnal et al for RBM5 (Bonnal et al, 2008). Although it would be difficult to formally disentangle such effect from its repression of intron splicing, the observation that RBM10 binding at downstream 3′ splicing sites exerts in general stronger effects than that at the immediately flanking ones (Fig 2E) implicated a possible dual function of RBM10 binding at 3′ splicing sites, *i.e*. repression of intron splicing and facilitation of the splicing site pairing.

### Functional investigation of an *in-frame* deletion of *RBM10* identified in a patient with TARP syndrome

**Figure 6:**
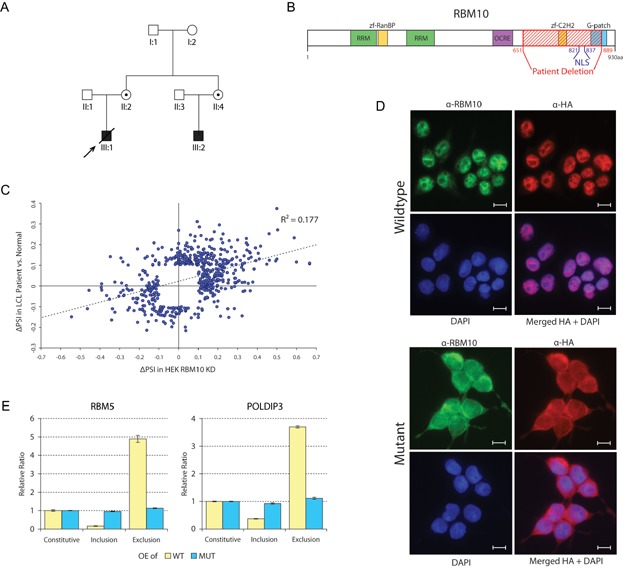
In-frame deletion of RBM10 identified in a family afflicted with multi-organ malformation Pedigree of the family.RBM10 protein domain structure, the deletion removed the second zinc finger domain, a portion of the G patch domain and a nuclear localization signal (NLS).Splicing difference (ΔPSI) between lymphoblastoid cell lines (LCLs) derived from the patient and those from healthy controls were correlated with splicing changes induced by RBM10 KD in HEK293.Subcellular localization of wild type and mutant RBM10 detected by antibody against RBM10 (a-RBM10) or that against HA tag. Co-staining with DAPI showed that in contrast to the nuclear localization of wild type, RBM10 mutant predominantly localized in the cytoplasm. Scale bar: 10 µm.Splicing changes of the two cassette exons in HEK293 cells upon overexpression (OE) of RBM10 wild type or mutant were measured by qPCR. The exclusion and inclusion levels were normalized based on constitutive exon expression.

Nonsense and frame-shift mutations in *RBM10* have been identified to be causative for TARP syndrome (Johnston et al, 2010). More recently, during a screen of >400 index patients from families with X-linked intellectual disability (ID; Kalscheuer et al., manuscript in preparation), a deletion of 1292 nt (ChrX: 46929367–46930658 bp, UCSC genome browser hg18) in RBM10 were found in a German family (Fig 6A and Supporting Information Fig S6A). Apart from severe ID, the two patients also suffered from multiple congenital malformations and their leading pathologic phenotypes overlapped with TARP syndrome (see Supporting Information and Table S7 for detailed reports of clinical findings). Based on the annotated gene structure, the deletion spanned six exons, but appeared not to disrupt the open reading frame. We performed RT-PCR and Western blot on the lymphoblast cells deriving from the patient, and demonstrated that the in-frame deletion did not induce nonsense mediated decay, which would otherwise degrade the truncated RNA and reduce protein levels (Supporting Information Fig S6B and C). The deletion removed 239 amino acids (651–889 amino acid at NP_005667), including the second zinc finger domain and a portion of the G patch domain (Fig 6B).

To understand how this *in-frame* deletion in *RBM10* could contribute to disease phenotype, we examined in more detail the sequence of the deleted fragment and identified a potential nuclear localization signal (NLS) within the deleted sequence (821–837 aa; Fig 6B and see also Supporting Information Supplementary Methods). To determine whether this finding was functionally relevant, we compared the subcellular localization of wild type RBM10 with that of the mutant. In contrast to the nuclear localization of wild type, RBM10 mutant predominantly localized in the cytoplasm (Fig 6D), consistent with the loss of NLS. This finding suggests that the deletion might result in loss of nuclear functions of RBM10. To investigate the impact of this deletion on gene expression especially splicing pattern, we performed RNA-seq on lymphoblastoid cell lines (LCLs), derived from the patient carrying the mutation and from healthy controls, respectively. We determined the changes in gene expression and splicing pattern using the same strategy applied on HEK 293 cells (Supporting Information Table S3 and S4). In total, we identified 206 and 102 exons showing enhanced inclusion and exclusion (|*Z*_PSI_| ≥ 3, |ΔPSI| ≥ 10%) in the patient derived LCLs, respectively. Intriguingly, the splicing changes observed here correlated well with changes induced by RBM10 KD in HEK293 (Fig 6C). This observation indicated that RBM10 mutant lost its function in splicing regulation, in accordance with the observed change in subcellular localization. To further validate the functional impact of the mutant, we overexpressed RBM10 in HEK 293 cells. As shown in Fig 6E, for the two cassette exons, the OE of the mutant could not induce the same splicing changes as that of the wild type.

## DISCUSSION

RBM10 has been characterized *in vitro* as an RNA-binding protein and identified as a component of spliceosome complex. However, its putative role in splicing regulation has not been established. In this study, our transcriptome-wide analysis of RBM10 binding profile as well as changes in splicing pattern induced by RBM10 perturbation provided experimental evidence supporting its role as a novel splicing regulator. Using PAR-CLIP, we identified thousands of consensus binding sites, 51% of which are located in the introns, with a significant enrichment in the vicinity of splicing sites. Using RNA-seq, we identified hundreds of exons, the splicing pattern of which was significant changed upon increasing or decreasing cellular RBM10 abundance. An RNA splicing map that associated RBM10-binding profiles with those observed splicing changes yielded a mechanistic model underlying RBM10 mediated splicing regulation.

Nonsense and frame-shift mutations in RBM10 have been identified to cause TARP syndrome (Johnston et al, 2010). In this study, in the two male cousins with congenital multi-organ malformation, we identified an *in-frame* deletion in RBM10, which removed the NLS of the protein and thereby largely disrupted its nuclear function. Whole-mount *in situ* expression analysis of the murine Rbm10 has shown that the gene was expressed during embryonic development in a pattern consistent with the human malformations observed in TARP syndrome (Johnston et al, 2010). Therefore, due to the loss of function of RBM10, our patients as well as the previously reported TARP patients would suffer from molecular defects in those tissues expressing critical amount of RBM10 during development. Given that RBM10 regulates many genes and possibly different sets of target genes in different tissues, it is likely that multiple targets would contribute to the phenotype.

Indeed, among the genes with splicing pattern regulated by RBM10, some have been implicated in the TARP syndrome associated anomalies (Supporting Information Table S4). For example, *DNML1* has found to be mutated in the patients with microcephaly and optic atrophy, overlapping features of TARP syndrome (Waterham et al, 2007). Mutations in *CEP290* could cause Joubert syndrome, a heterogenous ciliopathy characterized by cerebellar vermis hypoplasia and severe ID, two clinical findings also typical for TARP syndrome (Sayer et al, 2006). Another interesting gene, *CASK*, once mutated could lead to brain anomalies similar to the patients reported here (Najm et al, 2008). Finally, great phenotypic overlap is also noted to individuals with mutations in the *PIGN* gene, which lead to a syndromic entity characterized by hypotonia, seizures, neonatal hypotonia, lack of psychomotor development and dysmorphic features, associated with cardiac, urinary and gastrointestinal malformations (Maydan et al, 2011). Among the differentially expressed genes upon RBM10 perturbation, there are also candidate genes causing entities with overlapping features. For example, *ECEL1* is a gene responsible for distal arthrogryposis type 5D with similar limb anomalies observed in our patients (McMillin et al, 2013). Mutation in *LHB* gene could causes hypogonadism, which is also manifested in individuals with TARP syndrome (Weiss et al, 1992).

On one hand, the leading pathologic phenotypes observed in our patients largely overlapped with TARP syndrome (see Supporting Information for the discussion of clinical findings), indicating many, if not most of the TARP associated malformations resulted from loss of RBM10 nuclear function, *i.e*. regulation of exon skipping. On the other hand, comparing with typical TARP patients, our patients are relatively milder affected. Indeed they are the eldest patients reported so far. Given that only nonsense and frame-shift mutations in RBM10 have been reported in TARP patients, it is tempting to speculate that the mutant RBM10 in our patients might retain either some residue nuclear function or other unknown functions of the protein. Notably, a large number of RBM10-RNA interactions, especially those in exons, appeared not to be directly associated with splicing regulation. Whether such interactions hold other regulatory roles awaits further investigation.

More recently, in a large sequence analysis of lung adenocarcinomas, RBM10 was found to be frequently mutated and subject to recurrent nonsense, frame-shift or splice-site mutations (Imielinski et al, 2012). Interestingly, among the genes differentially spliced upon RBM10 perturbation in HEK293 cells, several were known factors associated with cancers (Supporting Information Table S4). Given the frequent observation of splicing deregulation in different types of cancers, it is plausible that RBM10 might also play an important role in cancers other than lung adenocarcinomas. Taken together, our study established RBM10 as an important regulator of AS, yielded a mechanistic model for RBM10-mediated splicing regulation and provided a starting point for the future functional characterization of RBM10 in different biological systems.

**The paper explained**

PROBLEM:

TARP syndrome is an X-linked inherited disorder leading to multiple organ malformation in affected males. Nonsense and frame shift mutations in *RBM10*, a gene encoding an RBPs, have been identified to cause TARP syndrome. Although the protein has been reported to associate with spliceosome complex, the exact molecular function of RBM10 is not clear.

RESULTS:

We combined photoactivatable-ribonucleoside-enhanced crosslinking and immunoprecipitation (PAR-CLIP) with massive parallel sequencing to identify RNA binding sites for RBM10 and observed significant RBM10-RNA interactions in the vicinity of splice sites. Using RNA-seq, we identified hundreds of splicing changes following perturbation of cellular RBM10 abundance. Integrative analyses of binding sites as well as splicing profile suggested a mechanistic model underlying RBM10-mediated splicing regulation, which could be subsequently validated by minigene experiments. Furthermore, we demonstrated the splicing defects in a TARP patient carrying an *in-frame* deletion in *RBM10*, which could be explained by disrupted function of RBM10 in splicing regulation.

IMPACT:

Our study for the first time established RBM10 as an important regulator of AS, presented a mechanistic model for RBM10-mediated splicing regulation and provided a molecular link to understanding a human congenital disorder.

## MATERIALS AND METHODS

### Cell lines

Stable HEK 293 T-REx Flp-In cell lines inducibly expressing FLAG/HA-tagged wild type and mutant RBM10, respectively were generated and maintained as described previously (Landthaler et al, 2008) with minor modifications (Supporting Information Supplementary Methods). Expression of FLAG-HA-tagged RBM10 was induced with 10 ng/ml doxycycline for 16 h.

### PAR-CLIP

The cells were labelled with 100 µM 4-thiouridine (4SU) and induced with 10 ng/ml doxycycline for 16 h. PAR-CLIP was performed as described previously (Hafner et al, 2010) with the following modifications. Cells were lysed in high salt lysis buffer (50 mM Tris–HCl pH 7.2, 500 mM NaCl, 1% NP40, 1 mM DTT, complete protease inhibitor (Roche)). For the second RNase T1 digestion, 10 U/µl RNase T1 and 5 min incubation was used. PAR-CLIP libraries were sequenced 1 × 50 cycles using Illumina HiSeq following the standard protocol. Detailed procedures see Supporting Information Supplementary Methods.

### RBM10 knockdown

siRNA (Applied Biosystems, s15747) against RBM10 was reverse transfected at a final concentration 20 nM with lipofectamine RNAiMAX (Invitrogen) in HEK293 T-REx Flp-In cells. Controls were treated with only transfection reagents. Cells were harvested 48 h after transfection, respectively. Total RNA was extracted using Trizol (Invitrogen) and the quality was assessed by Agilent Bioanalyser according to the manufacturer's instructions. The KD efficiency was assessed by qPCR and Western blot.

### RBM10 overexpression

Stable HEK293 T-REx Flp-In cells inducibly expressing FLAG-HA-tagged RBM10 was induced with 10 ng/ml doxycycline for 16 h. Control was treated with equal amount of medium. Total RNA was extracted using Trizol (Invitrogen) and the quality was assessed by Agilent Bioanalyser according to the manufacturer's instructions. The OE efficiency was assessed by qPCR and Western blot.

### mRNA sequencing

mRNA sequencing was performed using 1 µg total RNA. Briefly, poly (A) RNA was isolated by two rounds of oligo (dT)_25_ Dynabeads (Invitrogen) purification. Purified poly (A) RNA was fragmented at 94°C for 3.5 min using 5× fragmentation buffer (200 mM Tris–acetate, pH 8.1, 500 mM KOAc, 150 mM MgOA). The fragmented RNA was precipitated and converted to first strand cDNA using random hexmer primer and Supescript II (Invitrogen), followed by second strand cDNA synthesis with *Eschericcia coli* DNA pol I (Invitrogen) and RNAse H (Invitrogen). Then the paired-end sequencing library was prepared and sequenced on Illumina HiSeq for 2 × 100 cycles following the standard protocol.

### qRT-PCR

Total RNA was treated with TURBO DNase (Ambion) following the manufacturer's protocol. Reverse transcription was performed using 1 µg of DNase treated total RNA, random hexamer and Superscript III reverse transcriptase (Invitrogen) according to manufacturer's protocol. First stranded cDNA was diluted 1:20 and 2 µl was used as template in a 20 µl qPCR reaction system. qPCR was carried out using SYBRGreen Masrermix I (Roche) on LightCycler 480 (Roche) according to manufacturer's instructions. All assays were performed in triplicates. For expression quantification, the average fold change was calculated by normalization to *GAPDH*. For exon inclusion or exclusion quantification, the relative ratios were calculated by normalization to corresponding constitutive exons. The sequences of all PCR primers were listed in Supporting Information Table S5.

### Western blot

Western blot was performed as described in Supporting Information Supplementary Methods. The following antibodies were used: rabbit polyclonal anti-RBM10 (Abcam, ab26046, 1:2000), mouse monoclonal anti-HA (Covance, MMS-101P, 1:4000), mouse monoclonal anti-FLAG (Sigma, F1804, 1:4000) and rabbit polyclonal anti-GAPDH (Santa Cruz, sc-25778, 1:2000). Secondary HRP-conjugated goat anti-mouse or human IgG (Santa Cruz) was detected with SuperSignal Kit (Thermo).

### Minigene experiments

To validate the direct effects of RBM10 intronic binding on exon skipping, RBM10 protein was fused upstream of a modified PUF domain (PUF3-2) with specific and high affinity RNA recognition sequence (UGUAUGUA, *i.e*. A6G) as previously reported (Wang et al, 2009). The plasmid expressing the RBM10-PUF fusion protein was generated by overlapping PCR as previously described (Heckman & Pease, 2007) and then inserted into pcDNA3.1D/V5-His-TOPO expression vector (Invitrogen). Five hundred nanograms of splicing reporter (pZW2C-A6G) containing the PUF3-2 recognition sequence (Wang et al, 2009) was transfected alone, or cotransfected with 100 ng of RBM10-PUF or PUF expression vector, respectively into HEK293T cells in 12-well plate. After RT-PCR, the expression level of the two isoforms including or excluding the cassette exons was measured by Bioanalyser DNA 1000 chip (Agilent). All the PCR primers were listed in Supporting Information Table S5.

### PAR-CLIP sequencing data analysis

The PAR-CLIP reads were processed as described before (Lebedeva et al, 2011). In brief, the reads were aligned to the human genome (UCSC Genome Browser, hg19) allowing at most one mismatch, or indel of one nucleotide. Uniquely mapped reads were overlapped to define binding clusters. For each cluster, the preferred crosslinking position was defined as the site with the highest number of T to C conversions. Based on the binding clusters identified in the two biological replicates, a consensus binding cluster was defined as a pair of clusters from the two replicates, if preferred crosslinking site of one cluster from one replicate was located within the other cluster from the other replicate and vice versa. The preferred crosslinking site in the first replicate was used as that of the consensus cluster.

### RNA-seq data analysis

The RNA-seq reads were mapped with at most two mismatches to the human genome reference (UCSC genome browser hg19) and a set of sequences consisting of all possible junctions between the exons of each Refseq gene. The expression level of a gene was calculated as RPKM values by dividing the number of reads which could be mapped to the exons or exon–exon junctions of this gene by its cumulative exon length (in kb) and the total number of mappable reads (in million).

For each of the internal exon *E*, we computed percent splicing in value, PSI = *e*_in_/(*e*_in_ + *e*_out_), where *e*_in_ denotes the number of reads which could only be mapped to *E* or exon junctions containing *E* with an overlap of at least 6 bp, and *e*_out_ denotes the number of reads which could be mapped only to exon junctions skipping *E* and overlap with both exons by at least 6 bp. The internal exons with its PSI value between 0.02 and 0.98 in at least one data set were defined as cassette exons.

For each intron *I*, we computed percent intron retention value, PIR = *i*_in_/(*i*_in_ + *i*_out_), where *i*_in_ denotes the number of reads which could only be mapped to junctions between *I* and adjacent exons, and overlap with *I* by at least 6 bp, and *i*_out_ denotes the number of reads which could be mapped only to exon–exon junctions skipping *I* and overlap with both exons by at least 6 bp.

To estimate the significance of the change in gene expression level, PSI or PIR upon RBM10 KD and OE, we applied a *Z*-value transformation, *i.e*. divided Δlog_2_ RPKM/ΔPSI/ΔPIR by a local standard deviation which we computed using a sliding window approach as following. After sorting the exons according to the total number of reads used for computing the RPKM/PSI/PIR values (*e.g*. in the case of PSI, it is the sum of *e*_in_ and *e*_out_ from both OE/KD and control), we calculated for each data point the standard deviation of the respective values inside a window consisting 1% genes/exons/introns. The local standard deviations were then smoothed using loess regression before we used them for calculating *Z* values.

We then estimated false discovery rates using the rank product method (Breitling et al, 2004). For each independent replicate, the genes/exons/introns were ranked according to the respective *Z* values and the ranks obtained in the replicates were multiplied for each gene/exon/intron. The number of genes/exons/introns expected to have a given rank product by chance was estimated using random permutations of the rank lists.

The enrichment and annotation of functional categories in the set of genes with significant splicing changes or differentially expressed upon RBM10 perturbation was computed using the Database for Annotation, Visualization and Integrated Discovery (Huang et al, 2008, 2009).

## For more information

The raw sequencing data has been deposited in NCBI GEO database (Accession number: GSE44976). The PAR-CLIP data could also be accessed at doRiNA database (Anders et al, 2012):

http://dorina.mdc-berlin.de/rbp_browser/dorina.html

## Supplementary material

As a service to our authors and readers, this journal provides supporting information supplied by the authors. Such materials are peer reviewed and may be re-organized for online delivery, but are not copy-edited or typeset. Technical support issues arising from supporting information (other than missing files) should be addressed to the authors.
