# Supplementary material for: Integrative analysis revealed the molecular mechanism underlying RBM10-mediated splicing regulation
Source: EMBO Mol Med. 2013 Aug 22;5(9):1431–42. doi: 10.1002/emmm.201302663 (PMC3799496; doi:10.1002/emmm.201302663)
Supplement: Supplementary file 1 [file emmm0005-1431-SD1.pdf]

EMBO
Molecular Medicine

Integrative analysis revealed the molecular mechanism underlying RBM10-mediated splicing regulation

Yongbo Wang, Andreas Gogol-Döring, Hao Hu, Sebastian Fröhler, Yunxia Ma, Marvin Jens, Jonas Maaskola, Yasuhiro Murakawa, Claudia Quedenau, Markus Landthaler, Vera Kalscheuer, Dagmar Wieczorek, Yang Wang, Yuhui Hu and Wei Chen

Corresponding author: Wei Chen, Max Delbrück Center for Molecular Medicine

Review timeline:

Submission date:	20 February 2013
Editorial Decision:	26 March 2013
Revision received:	07 June 2013
Editorial Decision:	06 July 2013
Accepted:	11 July 2013

Transaction Report:

(Note: With the exception of the correction of typographical or spelling errors that could be a source of ambiguity, letters and reports are not edited. The original formatting of letters and referee reports may not be reflected in this compilation.)

Editor: Roberto Buccione

1st Editorial Decision 26 March 2013

Thank you for the submission of your manuscript to EMBO Molecular Medicine. We have now heard back from the three Reviewers whom we asked to evaluate your manuscript.
You will see that two Reviewers are more supportive of you work while one is quite negative. Nevertheless, all three raise significant issues that question the conclusiveness of the results thus preventing us from considering publication at this time. I will not dwell into much detail, as the evaluations are detailed and self-explanatory. I would like, however, to highlight a few main points.

Reviewer 1 is mainly concerned about the medical relevance of the observations. Specifically, s/he points to the incomplete characterisation of the patient whose cells were used. In addition, Reviewer 1 would like you to consider the potential consequences of alteration of splicing, due to RBM10, of other genes involved in congenital anomalies. S/he also notes the imprecise citation of previously reported information and other issues that require your action.

Reviewer 2 is especially concerned with overall data significance, completeness and quality and provides a detailed explanation and list of required remedies; I will just focus on the main points. Firstly and similarly to Reviewer 1, s/he feels that the medical angle of the study (which for EMBO Molecular Medicine is of high importance) is not sufficiently discussed and integrated. In this respect, I agree with Reviewer 1's assessment that relevant medical expertise might be useful in revising the manuscript. Reviewer 2 also notes that for the PAR-CLIP part, the motif analysis requires more explanation and further analysis. Also, s/he is of the opinion that the results of the mini-gene experiments have been overestimated/overstated; Remedies are suggested in this respect too. Reviewer 2 also points to flawed TARP syndrome analysis, including the interpretation of the consequences of the patient deletion. This Reviewer also lists many other critical points that require your action.

Reviewer 3 also points to technical flaws and issues of overall data significance and completeness. The many critical issues mentioned include the quality of controls. Again, although I will just focus on the main points, all items require your attention and action. Similarly to Reviewer 2, Reviewer 3 notes that the PAR-CLIP part requires extensive clarification including experimentation where necessary and is also concerned about the data on the RBM10 binding site clusters. Another item of strong concern, again in accord with Reviewer 2, is the outcome of the mini-gene experiments, which appear to have multiple criticalities. Finally, in a recurrent theme, Reviewer 3 is not satisfied with the connection to TARP syndrome. S/he also lists other experimental shortcomings and suggests a number of improvements that require action to increase the overall quality of data and presentation.

Considered all the above, while publication of the paper cannot be considered at this stage, we would be prepared to consider a substantially revised submission, with the understanding that the Reviewers' concerns must be fully addressed with additional experimental data where appropriate and that acceptance of the manuscript will entail a second round of review.

Since the required revision in this case appears to require a significant amount of time, additional work and experimentation and might be technically challenging, I would therefore understand if you chose to rather seek publication elsewhere at this stage. Should you do so, we would welcome a message to this effect.
Please note that it is EMBO Molecular Medicine policy to allow a single round of revision only and that, therefore, acceptance or rejection of the manuscript will depend on the completeness of your responses included in the next, final version of the manuscript.

As you know, EMBO Molecular Medicine has a "scooping protection" policy, whereby similar findings that are published by others during review or revision are not a criterion for rejection. However, I do ask you to get in touch with us after three months if you have not completed your revision, to update us on the status. Please also contact us as soon as possible if similar work is published elsewhere.

***** Reviewer's comments *****

Referee #1 (Comments on Novelty/Model System):

The in vitro studies use a combination of novel approaches. The studies performed on patient derived lymphoblasts are unique, few patients with mutation in RBM10 have been identified.

Referee #1 (Remarks):

This very well written and illustrated paper describes the effect of RBM10 on splicing of cassette exons and identifies a number of genes differentially affected by overexpression or knock down of RBM10. The laboratory studies are novel and very relevant.
A weakness of this paper is the lack of complete medical context. While there is a brief mention of the relevance to cancer, and the cancer genes are highlighted in the supplementary table, the more relevant primary context of embryologic development is not appropriately addressed. This major weakness should be addressed by asking the clinicians, who identified the patients on whom this work is based, to contribute to the written manuscript.
In the introduction you state the "100% pre- or postnatal lethality in affected males". This is wrong, as long term survival has been reported {Gripp et al., American Journal of Medical Genetics 2011}.
The description of the patient from whom the cell line was derived is insufficient. Is this patient alive or deceased? Is the cousin indicated on the pedigree alive or deceased? What are the typical malformations seen in TARP, and what major malformations were present in the patient whose cells you used?
It is clear from the data presented here that RBM10 affects splicing of numerous genes. Some of these genes listed in the supplementary table (CASK; TBX3; CREBBP; FANCA; POMT1 to name a few obvious examples) are known to be causally involved in congenital anomalies. This should be discussed. The genes known to be associated with congenital anomalies should be highlighted in the table (in addition to cancer- associated genes). Can any of these genes, by virtue of their abnormal splicing, result in the malformations seen in TARP syndrome?

While I realize that this is not the focus of this particular report, this is the context in which the work is relevant to human development and medicine. A clinicians' input in this manuscript is necessary.

Minor issues:
page 8: "anti-correlation"- do you mean inverse correlation?
The term "mental retardation" is not used in professional publications any longer; use "intellectual disability" instead.
Define TARP at its first use. What do the letters stand for?
page 14; middle paragraph: the sentence "This suggested this motif..." does not make sense as written.
"Acknowledgement" is misspelled.

Referee #2 (Comments on Novelty/Model System):

The model systems used were appropriate for the biochemical requirements of the study. The technical quality of the paper can be vastly improved by appropriate informatic controls (as pointed out in remarks below). The novelty is medium as mechanistically nothing was really teased apart.

Referee #2 (Remarks):

In the manuscript entitled "Integrative analysis revealed the molecular mechanism underlying RBM10-mediated splicing regulation" Wang et al. perform PAR-CLIP to identify genome-wide binding sites for the RBM10 protein, and perform RBM10 KD and overexpression experiments followed by RNA-seq analysis to identify RBM10 dependent splicing changes. The authors then use the genomics data to try to characterize the mechanisms of RBM10 action based on correlative analyses between RBM10 binding and alternative isoform usage. Finally, the authors perform a limited analysis of an apparent NLS deletion RBM10 mutation associated with human TARP syndrome, finding that this patient sample shares the alternative splicing profile of the RBM10 knockdown.

Although seemingly promising, the manuscript lacks a clear and compelling biological story that is supported with solid evidence, and seems to be missing many more detailed analyses to make the results more convincing. The CLIP and RNA-seq experiments will provide datasets for further research on RBM10, and the proposed link between RBM10 and the U2 snRNP is interesting (although not very well explored experimentally). However, the RBM10 binding mechanism remains unclear (as no motif is identified from the intronic binding sites, and the GAAGA motif identified from exonic binding sites apparently didn't validate in gel shift assays), and the effect of RBM10 binding, even in the limited minigene assays presented, is weak (~5% effect on exon inclusion). In addition, while the majority of the manuscript focuses on intronic RBM10 binding, the profile in Figure 2a is significantly shifted towards exonic binding (~40% exonic vs 50% intronic) as opposed to the whole human genome (less than 1:5 exonic to intronic), suggesting that the exonic binding sites are more likely to be biologically relevant.

Similarly, the TARP syndrome section feels a bit tacked on; it is interesting (though perhaps not surprising) that a patient sample with an RBM10 NLS deletion would resemble (at the splicing level) RBM10 knockdown, but it remains unclear to what degree this altered splicing pattern actually leads to phenotypic effects associated with the disease. As the authors don't discuss the degree to which gene expression is altered in these samples, it's not clear whether altered gene expression or altered splicing is the major component of TARP syndrome in this individual.

As a general comment, the supplemental figures need to be higher resolution (as they're impossible to read as presented here).

More detailed comments are below:

PAR-CLIP analysis:
• For the binding near exons (Fig 2b), there seems to be a control line missing. A shuffled control or even another protein's PAR-CLIP dataset would make the results for RBM10 more convincing.

• In Figure 1a, I assume that UTRs are included in the exon annotations - what does the distribution look like if you separate them from exons? Is there any UTR enrichment?
• The motif analysis seems to have been done in a very specific way in order to acquire some sort of enrichment. I missed any description of the algorithm used for motif finding other than 'pentamer enrichment' - was the GAAGA motif most significantly enriched, greatest fold-enriched, etc? A statistical test should also be included for significance of enrichment in exons and in the vicinity of both 5'and 3' splice sites of the introns. The authors also do not describe their definition of strong/medium/weak binding sites. I'm also confused by the apparent periodicity of pentamer frequencies in 2c - is there an explanation for why this might be observed? Or is this an artifact of some unspecified windowing or normalization procedure?
• Fig 2d seems unnecessary as a main figure or even a supplemental figure, as it is a very weak result.
• The discussion includes mention of a gel-shift assay that failed to detect direct binding of RBM10 to the GAAGA motif described in figure 2c. This result needs to be presented in the results section during the discussion of figure 2c, as it has significant implications for interpretation of figure 2c. It also needs to be further discussed, as at the end of the paper it is unclear whether the authors believe that this reflects an RBM10 motif or rather a motif of some unspecified additional regulator that RBM10 associates with.
• The inference of U2 snRNA binding by PAR-CLIP read density in Figure 2e is interesting - for the second part of figure 2e, it would be helpful to include the data (read counts / base), as it's unclear whether the two indicated positions are most enriched or are the only positions observed to have the T->C crosslink transitions.

RBM10 OE/KD RNA-seq:
• The authors go straight to altered splicing upon RBM10 KD and OE - are there significant alterations at the gene expression level? I recognize that the focus of this paper is on alternative splicing regulation by RBM10, but the scale to which RBM10 manipulation generally effects gene expression would be valuable (as well as information on other RNA binding proteins significantly altered in the RBM10 KD/OE experiments).
• In Supplemental Fig. 2a, the western blot for the RBM10 KD and control sample needs to be shown together on the same western blot as done in Supp Fig 2b. As it is, it appears that the authors pasted together two lanes from potentially different gels, which may or may not show an actual KD at the protein level. Quantification (particularly of the knockdown western blot) would also be ideal, as it appears that GAPDH is also lower in the KD sample.
• It seems that the overlap of events changed in the OE and the KD is 306, but what about the events that do not overlap (not in both the OE and KD experiment)- is there anything interesting with those? Are these just false positives or were they just missed in one of the experiments? Can they be validated in both conditions?
• The authors report 17 events that validated via qPCR - however, it is difficult to determine from the data shown how representative these events are, as 11 of the 17 have >~2-fold changes in PSO whereas the majority of events in figure 3a have deltaPSI <|0.2|. Additionally, detecting splicing changes by qPCR requires a fair amount of normalization calculations, but these validations can be done more simply with RT-PCR and running agarose gels. Can the splicing changes be detected this way or were they only detectable by qPCR?
• Figure 3c shows a track labeled "ctrl" - is this the control for the OE or the KD? (The authors list independent control datasets for both experiments - hopefully separate controls were used in all splicing experiments throughout the paper. This should be made explicit and corrected if not).
• Splicing maps are made with the RBM10 OE data, what do they look like with the KD data - hopefully the same.
• What fraction of the events that change are associated with RBM10 binding sites? Conversely, the PAR-CLIP experiment identified many (~88k) binding sites - what percent of binding sites are associated with regulated targets?

RBM10 splicing models:
• The minigene experiments seem like a nice idea, but I unfortunately I don't think the results are as convincing as claimed. In Figure 4, the authors state that "These data provide unequivocal support to our hypothesis that RBM10 binding ... would facilitate the skipping of cassette exons", but the lack of technical qPCR error bars as well as some sort of statistical test to show whether the observed changes are significant conflict with such a strong statement. Additionally, the effect on splicing for three of the four mutations made in Fig 4b/c is extremely small (~5% or less), and the data shown

only reflects the change in exon exclusion between OE and control. I was curious as to whether the mutations performed show significantly altered PSO rates in the control (or over-expression) by themselves, and to what the rates of exon inclusion for these events typically are (i.e., if they were 90% excluded in the control then a small effect upon RBM10 OE would be not surprising). Additionally, it would be ideal to perform the direct experiment (forced RBM10 association with intronic loci, e.g. by MS2 tagging) to prove that RBM10 recruitment to an intron will alter splice site choice.
• P-values are missing for Fig. 5d. The strength of splicing sites distal to the cassette exons does not seem significantly stronger than that of those immediately flanking the exons, and in general the effect size here seems to be very slight.
• It looks like fig 6b should be recreated by the authors, seems like it was taken from another publication.

TARP syndrome analysis:
• Intriguingly, the splicing changes observed here correlated well with changes induced by RBM10 KD in HEK293 (Fig. 6c). What about compared to the OE experiment?
• Supplemental figure 3 needs better quantification - it is clear from the figure that the mutated RBM10 is not silenced, but it is difficult to tell whether the expression is actually unchanged from the results shown. Figure 6c also needs statistics - what is the correlation between the two samples?
• Figure 6c-e are used to propose that this patient deletion acts as an RBM10 deletion through loss of nuclear expression. However, the deletion includes not only the NLS but also additional sequences. Figure 6e would be strengthened by mutating either only the NLS, or attaching a normal NLS to the mutated RBM10 to show that the molecular phenotypes observed in this patient are characteristic mis-localization of RBM10 and not also loss of function through deletion of additional domains.

Discussion comments:
• Overall, the discussion needs to be rewritten to put the results of the paper in context. The idea the RBM10 works with other RBPs needs to be presented sooner than the discussion, otherwise the results section makes no sense in this context - for example, it is not until the discussion that the authors imply that RBM10 does not seem to bind the identified motifs by itself. Similarly, the TARP section needs a better description of how it fits with the results in the rest of the paper - if RBM10 loss of function causing TARP is previously known, is the novelty the validation of the individual deletion patient as actually losing nuclear localization? Or that the loss of nuclear localization of RBM10 resembles RBM10 knockdown?
• The statement "Whole-mount in situ expression analysis of the murine Rbm10 has shown that the gene was expressed during embryonic development in a pattern consistent with the human malformations observed in TARP syndrome" is missing a reference.
• The discussion refers to two experiments that need to be incorporated into the main text, as they are not mentioned anywhere in the manuscript before the discussion - one experiment (an in vitro binding RBM10 experiment) that is not shown in any figures and only mentioned in the supplemental methods, and discussion of the effect of exonic RBM10 binding (figure 7), which is also not presented in the results.

Minor comments:

-Breitling et al 2004 citation in the methods section is misformatted and not included in reference list
-typo in figure reference: "In agreement with our in vivo data, the skipping of the cassette exons was enhanced upon RBM10 OE (Fig. 54b)"
-Figure 5 figure legend is mislabeled as figure 4

Referee #3 (Comments on Novelty/Model System):

Technical quality
Major issues:

1. Filtering out potential contaminants. While most of the reads are derived from cross-linked RNA species (carry U to C transitions) , from supplementary figure 1a it is clear that there is successful RNA pull-down from the control samples that were not cross-linked. Such result indicates that the washing stringency may not have been sufficient to remove species of RNA retained non-specifically on the resin or bound through mediated interaction. Reads derived from such non-specifically interacting RNAs can clearly be identified in PAR-CLIP by the absence of U to C transition in the sequence. Were those reads removed from the set that was used to define the RBM10 binding site?
2. Binding site clusters. The authors need to provide more information on the cluster that were identified in the PAR-CLIP experiment:
(i) What is the distribution of the cluster sizes?
(ii) How many reads are forming a typical cluster and what is the read count distribution of the clusters? The relatively small overlap between the two experiments would indicate that most of the cluster are formed by relatively few reads.
(iii) Within each cluster, are there preferred cross-linking sites? This would be a strong indication of the presence of high affinity binding site.
3. RBM10 binding site sequence. The authors identify GAAGA as a pentamer that is enriched in the RBM10 binding site clusters that are located within exons. The absence of Uridine residue from the binding site is quite surprising considering that PAR-CLIP is reliant on cross-linking to U residues. It raises the possibility that the approach used to identify the binding site sequence is inadequate. In particular what was the rational to analyze separately the exonic and intronic binding sites? Do the authors expect RBM10 to bind to a different sequence in the introns compared to the exons? Was the GAAGA pentamer significantly enriched in the intronic binding sites? If not, than what is the evidence of sequence specific binding of RBM10 to RNA?
Alternatively it is possible that the sequence recognized by RBM10 does not contain uridines that can be cross-linked to the protein. This would mean that PAR-CLIP is not an adequate experimental approach for identifying RBM10 binding sites and the standard CLIP approach, that uses short wavelength UV light to crosslink to unmodified RNA should be used instead.
4. Binding to U2. The finding that RBM10 binds to U2 snRNA is quite intriguing. How many reads were mapped to U2 compared to the reads mapped in protein coding genes. Considering the high abundance of the snRNP RNAs, is there enrichment of RBM10 cross-links to U2 that is statistically significant?
5. Correlation between RBM10 binding and effect on splicing. One strong aspect of the presented work is the availability of both RNA binding (PAR-CLIP) and exon inclusion data (RNA-seq). Surprisingly the authors do not provide information as to what fraction of the RBM10 regulated exons contain RBM10 binding sites. Good correlation between RBM10 binding and exon inclusion/skipping would strongly argue that the effect on splicing is specific to RBM10 and is not a secondary effect.
6. RNA splicing map. Why are the exonic sequences ignored in the RNA map (figure 3d)? This makes no sense considering that most of the RBM10 binding clusters are located in exons (figure 2b).
7. Minigene experiments (figure 4). There are multiple issues with the data presented on this figure:
(i) Disrupting the binding sites does significantly disrupt the effect of RBM10 on splicing, possible with the exception of Mut D5. This is a very strong argument that the authors did not identify the correct binding sites. This data not only does not "provide unequivocal support", but directly contradicts the authors conclusion that "...RBM10 binding in the vicinity of splice sites of flanking introns would facilitate the skipping of the cassette exons".
(ii) While the RBM10 effect on PUF60 splicing is mostly abolished in PUF60 Mut D5, this is by no means conclusive evidence that the site disrupted in Mut D5 is the cis-acting element that is recognized by RBM10. The mutation may have disrupted sequence element recognized by a different protein that is required for regulation of the alternative exon. The authors need to show that placing the RBM10 binding sites in vicinity of a heterogonous alternative exon confers regulation by RBM10.
(iii) There is no statistical analysis to show how significant the changes in exon inclusion are and what is the variability of the assay.
(iv) The authors need to show the sequences of the wild type and mutated binding sites.
(v) the Authors need to show gel images of the RT-PCR reactions.
8. The authors convincingly show the effect of RBM10 mutations in patients on alternative splicing. However RNA binding proteins frequently multitask and regulate RNA stability and translation. It would be interesting to know if there are transcripts with altered abundance and the patient

lymphoblasts and after the RBM10 knockdown in HEK cells. Also does RBM10 bind to the mRNA UTRs, which are frequently involved in regulation of translation and RNA stability. The answers to these questions should already be in the PAR-CLIP and RNA-Seq data. The fact that the two patients presented in this study display milder phenotypes compared to the typical TARP syndrome, despite having impaired function of RBM10 in slicing would argue that the protein may have additional functions in the cytoplasm.
Minor issues:
1. Figure 1b. From the methods it is unclear why the values for the consensus clusters are higher than the values for the reads. If clusters are aggregates of reads, one would expect the cluster density to be less than the read density.
2. On Figure 2c the authors need to define what is considered to be "Strong", "Medium" and "Weak" binding site.
3. On Figure 3c showing the aligned PAR-CLIP reads, rather than a triangle will be more informative to the reader.

Novelty
Although association with the spliceosome raises the possibility that RBM10 regulates alternative splicing this has not been shown to date. Furthermore, the rich sequence data obtained in this study can provide significant insight into the mechanisms by which RBM10 regulates splicing.

Medical Impact
Mutations in RBM10 have been associated with developmental disorders. Furthermore, it is frequently mutated in certain types of cancer. Understanding its function may contribute to developing cancer therapies and prognostic markers.

Adequacy of the model system
The authors express epitope tagged RBM10 for the PAR-CLIP experiments. While this is acceptable, particularly in cases where good quality antibodies are not immediately available for the endogenous protein, the authors need to show that the levels of the expressed protein are comparable to those of the endogenous protein. Maintaining physiological protein levels is critical as over-expression may result in binding to low affinity sites on the RNA that are not occupied under normal conditions.

Referee #3 (Remarks):

RBM10 is an RNA binding protein that has been associated in several studies with the spliceosome. However, its function there has remained unclear. The work presented by Wang et al in this manuscript ascribes a function of RBM10 in splicing and more specifically in exon recognition. The authors use a combination of PAR-CLIP and RNA-seq to build an integrated model for RNA splicing regulation by RBM10. Wang et al also show that alternative splicing patterns in patients with TARP syndrome resemble those of RBM10 knockout cell lines, concluding that the splicing regulatory function of RBM10 is disrupted in the patients. The results of the presented work can potentially have a significant impact on our understanding of splicing regulation in organism development and human disorders including cancer.
While the quality of the raw data appears to be adequate the subsequent analysis leaves a lot to be desired (see the specific comments for details). The results as presented in the manuscript do not support the proposed RBM10 binding site sequence and the model for splicing regulation by RBM10. There are also some issues that need to be addressed in respect to the model system. In particular, the use of protein over-expression in the PAR-CLIP experiments may result in the identification of binding sites that are not normally occupied by RBM10. If these deficiencies are adequately addressed the work by Wang et al will without doubt have significant impact.

1st Revision - authors' response 07 June 2013

We would like to thank the three referees for carefully reviewing our manuscript and appreciate the constructive critique raised by the referees. In what follows, we address all aspects that required correction or clarification.

Summary of important changes in the revision:

1. Following the suggestion of all three referees, we asked Dr. Dagmar Wieczorek, the clinical geneticist, who has been taking care of the family, to add the clinical data and provide her opinion on the genes regulated by RBM10. In short, we added a detailed case report of the affected cousins (see Supplementary Information) and a table summarizing the phenotype comparison between our patients and TARP patients reported before (Supplementary table 7). We added a chapter concerning the relevance of the genes regulated by RBM10 in causing phenotypes overlapped with our patients and/or TARP patients in the discussion part.

2. Following the suggestion of referee #2 and referee #3, we performed further analysis of our PAR-CLIP and RNA-seq data. In the revised manuscript, we added detailed clarification about RBM10 binding clusters, such as the distribution of length, number of PAR-CLIP reads and etc. We incorporated the appropriate control and statistical analysis when necessary. We added a chapter and a table (supplementary table 3) listing the genes differentially expressed upon RBM10 perturbation.

3. Following the suggestion of referee #2 and referee #3, we performed two additional minigene experiments in a heterologous context. 1) we inserted RBM10 binding sites into intronic location of a new splicing reporter, pZW2C; 2) we fused RBM10 with a modified pumilio domain, PUF3-2, which specifically recognizes an eight-nt sequence 'UGUAUGUA' with high affinity, thereby, we could tether RBM10 close to (18nt downstream) splicing sites of the cassette exon in another splicing reporter. Both minigenes could demonstrate that intronic binding of RBM10 near splice sites indeed could enhance exon skipping in a heterologous context.

4. Following the suggestion of referee #2, we re-organized the discussion part and moved the discussion of RBM10 exonic binding to the result part.

We believe that our additional experiments and computational analysis helped to substantially improve the quality of the paper and to address the requests made by the referees. We added two new authors who have been involved substantially in the revision.

Please find an exhaustive point-by-point response below. We are grateful that you can consider the manuscript for your journal.

Referee #1 (Remarks):

This very well written and illustrated paper describes the effect of RBM10 on splicing of cassette exons and identifies a number of genes differentially affected by overexpression or knock down of RBM10. The laboratory studies are novel and very relevant.
A weakness of this paper is the lack of complete medical context. While there is a brief mention of the relevance to cancer, and the cancer genes are highlighted in the supplementary table, the more relevant primary context of embryologic development is not appropriately addressed. This major weakness should be addressed by asking the clinicians, who identified the patients on whom this work is based, to contribute to the written manuscript.

We thank the referee for his/her appreciation of the novelty and relevance of our study, and thank him/her and other referees for pointing out the weakness of our manuscript. Following his/her suggestion, we asked Dr. Dagmar Wieczorek, the clinical geneticist, who has been taking care of the family, to add the clinical data and provide her opinion on the genes regulated by RBM10.

In the introduction you state the "100% pre- or postnatal lethality in affected males". This is wrong, as long term survival has been reported {Gripp et al., American Journal of Medical Genetics 2011}.

We thank the reviewer for this comment: All but one patient described before died pre- or post-natally. We corrected this in the revision. Indeed, the two cousins described here are the eldest individuals with TARP syndrome so far.

The description of the patient from whom the cell line was derived is insufficient. Is this patient alive or deceased? Is the cousin indicated on the pedigree alive or deceased? What are the typical malformations seen in TARP, and what major malformations were present in the patient whose cells you used?

We added detailed case report of the cousins (see Supplementary Information) and a table summarizing the phenotype comparison between our patients and TARP patients reported before (Supplementary table 7). The index patient, from whom the cell line was available, deceased, whereas the younger cousin is still alive. We update the pedigree to make this information clearer for the reader (see Figure 7A). In the Supplementary Information and supplementary table 7, we have listed all the major malformations present in reported TARP patients and/or our two patients.

It is clear from the data presented here that RBM10 affects splicing of numerous genes. Some of these genes listed in the supplementary table (CASK; TBX3; CREBBP; FANCA; POMT1 to name a few obvious examples) are known to be causally involved in congenital anomalies. This should be discussed. The genes known to be associated with congenital anomalies should be highlighted in the table (in addition to cancer- associated genes). Can any of these genes, by virtue of their abnormal splicing, result in the malformations seen in TARP syndrome?

We thank the referee for pointing this out. Following his/her suggestion, we added a chapter concerning this point to the discussion part. In the chapter, we focused on the genes implicated in the TARP syndrome associated anomalies and therefore did not discuss TBX3, CREBBP, FANCA and POMT1 because the reported phenotype caused by the mutation identified in these four genes did not overlap with the anomalies in TARP syndrome: TBX3 mutations are causative for ulnar mammary syndrome, which is characterized by posterior limb deficiencies and mammary gland hypoplasia amongst others. Both main clinical findings were not observed in the individuals with TARP syndrome. Mutations in the *CREBBP* gene cause Rubinstein-Taybi syndrome, which is a recognizable multiple congenital anomaly syndrome with intellectual disability (ID). The facial features and the broad thumbs and toes are very characteristic and different from TARP syndrome. *FANCA* is one of the genes associated with Fanconi anemia. There are overlapping clinical findings (pre- and postnatal growth failure, internal malformations, hearing loss), but radial or thumb anomalies and early bone marrow failure, two major features of Fanconia anemia, were not reported in the individuals with TARP syndrome. Dystroglycanopathies are caused by mutations of the *POMT1* gene. The clinical spectrum of anomalies is wide and thus overlapping with TARP syndrome. As results of muscle biopsies were not reported in the TARP individuals, one cannot exclude that there might be dystrophic changes, but the pattern of malformations in TARP syndrome seems to be different from the *POMT1* related disorders, e.g. Walker-Warburg syndrome.

While I realize that this is not the focus of this particular report, this is the context in which the work is relevant to human development and medicine. A clinicians' input in this manuscript is necessary.

Dr. Dagmar Wieczorek, the clinical geneticist, who has been taking care of the family, added the clinical data and provided her opinion on the genes regulated by RBM10.

Minor issues:
page 8: "anti-correlation"- do you mean inverse correlation?

Yes. We replaced the term.

The term "mental retardation" is not used in professional publications any longer; use "intellectual disability" instead.

Thanks for pointing this out, we corrected it.

Define TARP at its first use. What do the letters stand for?

TARP stands for Talipes equinovarus, Atrial septal defect, Robin sequence, and Persistent left superior vena cava. We now added the definition at its first appearance in the manuscript.

page 14; middle paragraph: the sentence "This suggested this motif..." does not make sense as written.

We changed the sentence now to "This suggested the motif might not represent the specific sequences recognized by RBM10".

"Acknowledgement" is misspelled.

Thanks for pointing this out, we corrected it.

Referee #2 (Remarks):

In the manuscript entitled "Integrative analysis revealed the molecular mechanism underlying RBM10-mediated splicing regulation" Wang et al. perform PAR-CLIP to identify genome-wide binding sites for the RBM10 protein, and perform RBM10 KD and overexpression experiments followed by RNA-seq analysis to identify RBM10 dependent splicing changes. The authors then use the genomics data to try to characterize the mechanisms of RBM10 action based on correlative analyses between RBM10 binding and alternative isoform usage. Finally, the authors perform a limited analysis of an apparent NLS deletion RBM10 mutation associated with human TARP syndrome, finding that this patient sample shares the alternative splicing profile of the RBM10 knockdown.

Although seemingly promising, the manuscript lacks a clear and compelling biological story that is supported with solid evidence, and seems to be missing many more detailed analyses to make the results more convincing. The CLIP and RNA-seq experiments will provide datasets for further research on RBM10, and the proposed link between RBM10 and the U2 snRNP is interesting (although not very well explored experimentally). However, the RBM10 binding mechanism remains unclear (as no motif is identified from the intronic binding sites, and the GAAGA motif identified from exonic binding sites apparently didn't validate in gel shift assays), and the effect of RBM10 binding, even in the limited minigene assays presented, is weak (~5% effect on exon inclusion). In addition, while the majority of the manuscript focuses on intronic RBM10 binding, the profile in Figure 2a is significantly shifted towards exonic binding (~40% exonic vs 50% intronic) as opposed to the whole human genome (less than 1:5 exonic to intronic), suggesting that the exonic binding sites are more likely to be biologically relevant.

Similarly, the TARP syndrome section feels a bit tacked on; it is interesting (though perhaps not surprising) that a patient sample with an RBM10 NLS deletion would resemble (at the splicing level) RBM10 knockdown, but it remains unclear to what degree this altered splicing pattern actually leads to phenotypic effects associated with the disease. As the authors don't discuss the degree to which gene expression is altered in these samples, it's not clear whether altered gene expression or altered splicing is the major component of TARP syndrome in this individual.

As a general comment, the supplemental figures need to be higher resolution (as they're impossible to read as presented here).

Following the suggestion of the referee, we performed further analysis of our PAR-CLIP and RNA-seq data. In the revised manuscript, we added detailed clarification about RBM10 binding clusters. We incorporated the appropriate control and statistical analysis when necessary. We added a chapter and a table (supplementary table 3) listing the genes differentially expressed upon RBM10 perturbation. In addition, to validate the impact of RBM10 binding on alternative splicing, we performed two additional minigene experiments in a heterologous context. Both minigenes could demonstrate that intronic binding of RBM10 near splice sites indeed could enhance exon skipping in a heterologous context. We admit that we did not detect sequence motif directly recognized by RBM10. However, given that many protein interaction partners of RBM10 are RNA binding

proteins (see Hegele A, et.al. Molecular cell 45: 567-580), it is plausible that RBM10 binds to RNA also indirectly via other interaction partners.

We agree with the referee, it appeared that RBM10 binding clusters were more enriched in exons than in introns (see also the table presented in the detailed point-by-point response below). However, please note the number of nucleotides in the genome corresponding to exonic or intronic regions was used as the background to calculate the enrichment. Given that the percentage of introns and exons represented in cellular RNA is very different from that in the genome, (i.e. the majority of cellular RNA derived from protein-coding genes are mature mRNAs containing no introns), and that the IP was performed on RNA instead of genomic DNA, such enrichment value should be treated more cautiously. As to the potential function of exonic binding, we observed that RBM10 binding at exonic GAAGA sites appeared to override the exon skipping effect of intronic binding. Except this, we tried, but failed to find any additional clear correlation between exonic binding and splicing changes induced by RBM10 perturbations. Comparing with the neighboring constitutive exons, the density of binding sites in the cassette exons was reduced. This was observed for all the cassette exons, the exons more excluded upon RBM10 OE, as well as the exons more included upon RBM10 OE, and could probably be explained by the fact that cassette exons are non-constitutive and are therefore not always present in all transcripts. The fact that there is no clear difference in exon binding between different groups of exons indicates that the exon binding alone might not regulate splicing. Having said these, we could not exclude the effect of exonic binding on other processes such as mRNA translation, which is beyond the scope of this study.

In the revised manuscript, we added a detailed case report of the affected cousins (see Supplementary Information) and a table summarizing the phenotype comparison between our patients and TARP patients reported before (Supplementary table 7). We added a chapter concerning the relevance of the genes regulated by RBM10 in causing phenotypes overlapped with our patients and/or TARP patients in the discussion part.

We increased the resolution of all supplementary figures, which are now in vector-based format.

We believe that our revision substantially improved the quality of the paper and addressed the requests made by the referee. Please find an exhaustive point-by-point response below.

More detailed comments are below:

PAR-*CLIP* analysis:
For the binding near exons (Fig 2b), there seems to be a control line missing. A shuffled control or even another protein's PAR-CLIP dataset would make the results for RBM10 more convincing.

We now added AGO2 PAR-CLIP dataset (the same PAR-CLIP experimental condition in the same cell line, i.e. HEK293) as control. As shown in Figure 2, compared with AGO2, RBM10 binding shows clear enrichment in the vicinity of both 5' and 3' splicing sites.

In Figure 1a, I assume that UTRs are included in the exon annotations - what does the distribution look like if you separate them from exons? Is there any UTR enrichment?

We now changed the pie chart of genomic distribution of RBM10 binding clusters, in which the exons were separated into coding sequence, 5' and 3' UTR (see Fig. 2A). Comparing with coding exons and the splicing sites, both 5' and 3' UTR show much less enrichment, see the table below,

		RBM10 Binding Sites		Nucleotides		Fold Enrichment
**Exons**		34,426	(39.1%)	70,480,509	(2.5%)	15.87x
	**5' UTR**	921	(1.0%)	11,843,639	(0.4%)	2.53x
	**CDS**	30,327	(34.5%)	34,759,483	(1.2%)	28.36x
	**3' UTR**	3,178	(3.6%)	23,877,387	(0.8%)	4.33x
**Introns**		45,673	(51.9%)	1,006,012,043	(35.2%)	1.48x

© EMBO

	**5' SS**	8,340	(9.5%)	26,221,461	(0.9%)	10.34x
	**Intron Center**	25,170	(28.6%)	953,551,249	(33.4%)	0.86x
	**3' SS**	12,163	(13.8%)	26,239,333	(0.9%)	15.07x
**Intergenic**		7,858	(8.9%)	1,782,182,170	(62.3%)	0.14x
**Total**		87,957	(100.0%)	2,858,674,722	(100.0%)	1.00x

Please note we used the number of nucleotides in the genome corresponding to different genomic regions as the background to calculate the enrichment. As explained above, given that the percentage of these regions represented in cellular RNA is very different from that in the genome, such enrichment value should be treated more cautiously.

The motif analysis seems to have been done in a very specific way in order to acquire some sort of enrichment. I missed any description of the algorithm used for motif finding other than 'pentamer enrichment' - was the GAAGA motif most significantly enriched, greatest fold-enriched, etc? A statistical test should also be included for significance of enrichment in exons and in the vicinity of both 5'and 3' splice sites of the introns. The authors also do not describe their definition of strong/medium/weak binding sites. I'm also confused by the apparent periodicity of pentamer frequencies in 2c - is there an explanation for why this might be observed? Or is this an artifact of some unspecified windowing or normalization procedure?

The motif analysis was done in a very simple and straightforward way. We determined the frequency of pentamers inside 40-nt windows around preferred crosslinking sites and compared them to the frequency around control sites. There is no clear sequence motif enriched in the intronic binding sites. The most abundant pentamer around RBM10 exonic binding sites was GAAGA, e.g. 25.3% of all RBM10 sites have a GAAGA motif in up to 20bp distance, whereas only 10.8% of the control sites have a GAAGA motif in up to 20bp distance (Fisher exact test p-value < 10-16). As shown in the figure below, other motifs with high enrichment are just slight deviants of GAAGA.

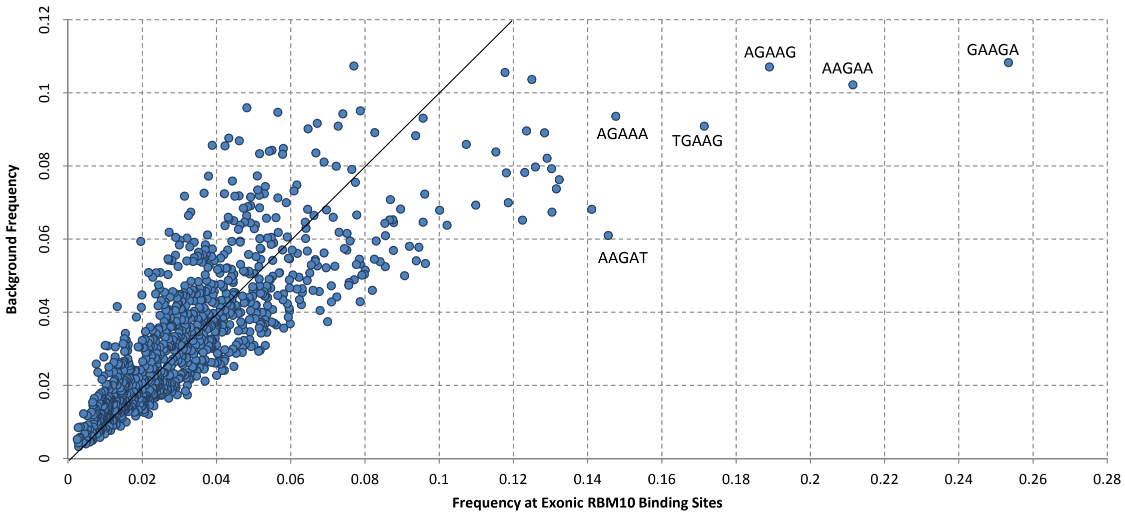

We now added the definition of strong/medium/weak binding sites in the method section. In brief, we sorted the binding clusters according to the number of reads spanning the binding site. The clusters were then binned into three groups of equal size.

The periodicity of pentamer frequencies is due to codon usage and the fact that we required a T at position 0.

Fig 2d seems unnecessary as a main figure or even a supplemental figure, as it is a very weak result.

We think Fig. 2D showed that although there is no sequence motif for intronic binding, there is still clear bias in nucleotide composition. Interestingly, such bias is similar between the binding at 5' and

3' splicing sites, although those sites have quite different background base composition, indicating the bias may represent the sequence preference of RBM10 binding.

The discussion includes mention of a gel-shift assay that failed to detect direct binding of RBM10 to the GAAGA motif described in figure 2c. This result needs to be presented in the results section during the discussion of figure 2c, as it has significant implications for interpretation of figure 2c. It also needs to be further discussed, as at the end of the paper it is unclear whether the authors believe that this reflects an RBM10 motif or rather a motif of some unspecified additional regulator that RBM10 associates with.

As the referee suggested, we now presented the gel shift assay (Supplementary Fig 1.G) and discussed the relevance of this finding in the result part, immediately following the motif identification part. We make it clear that we believe that RBM10 binds to the motif via other interaction partner(s).

The inference of U2 snRNA binding by PAR-CLIP read density in Figure 2e is interesting - for the second part of figure 2e, it would be helpful to include the data (read counts / base), as it's unclear whether the two indicated positions are most enriched or are the only positions observed to have the T->C crosslink transitions.

As the referee suggested, we included now another figure (Fig. 2E, up right panel) for the density of T-C conversion reads along U2. As shown in the plot, there are clearly two crosslink sites with most enriched T-C conversions, indicated in the figure below (Fig. 2E, low panel).

RBM10 OE/KD RNA-seq:
The authors go straight to altered splicing upon RBM10 KD and OE - are there significant alterations at the gene expression level? I recognize that the focus of this paper is on alternative splicing regulation by RBM10, but the scale to which RBM10 manipulation generally effects gene expression would be valuable (as well as information on other RNA binding proteins significantly altered in the RBM10 KD/OE experiments).

We now added the following paragraph describing the gene expression changes induced by RBM10 OE/KD in the result part.

"The gene expression level was estimated based on RPKM (reads per kilobase of exon per million mapped sequence reads, (Mortazavi et al., 2008), Material and Methods). At false discovery rate (fdr) < 0.05, 171 and 105 genes were found to be significantly upregulated and downregulated by at least 1.5 fold upon RBM10 KD (Figure S2F and Table S3), whereas 19 and 49 genes were upregulated and downregulated to the same level (fdr < 0.05, fold change ≥ 1.5) in response to RBM10 OE, respectively (Figure S2F and Table S3). Notably, the expression changes induced by KD and OE were not inversely correlated (Figure S2G)."

Among the gene differentially expressed under RBM10 OE/KD, 14 were RNA-binding proteins and five were known to be splicing regulators, which might have secondary effect on the splicing changes induced by RBM10 perturbation. We added this point in the result part.

In Supplemental Fig. 2a, the western blot for the RBM10 KD and control sample needs to be shown together on the same western blot as done in Supp Fig 2b. As it is, it appears that the authors pasted together two lanes from potentially different gels, which may or may not show an actual KD at the protein level. Quantification (particularly of the knockdown western blot) would also be ideal, as it appears that GAPDH is also lower in the KD sample.

We now included the original western blot in Supp Fig2 and showed the change at the protein level based on quantification of the signal intensity from the specific band (normalized to the level of GAPDH).

It seems that the overlap of events changed in the OE and the KD is 306, but what about the events that do not overlap (not in both the OE and KD experiment)- is there anything interesting with those? Are these just false positives or were they just missed in one of the experiments? Can they be validated in both conditions?

We believe the splicing changes that were only observed in either OE or KD, or those that were positively correlated between OE and KD, are true events. The major reasons for such a behavior include 1) The basal level of the exon inclusion in unperturbed HEK293 cells is too high or too low. In the former situation, the change induced by OE, i.e. exon skipping, could be very obvious whereas KD could hardly produce any further/additional exon inclusion. The opposite situation holds also true for those exons with too low basal level of exon inclusion. As shown in the figure below, compared with those exons showing splicing changes in both conditions, the exons with changes in only one condition have more extreme basal level of exon inclusion. 2) Those events represent secondary effects, which could result from the expression change of certain splicing regulators. As discussed above, the expression changes were not inversely correlated between KD and OE.

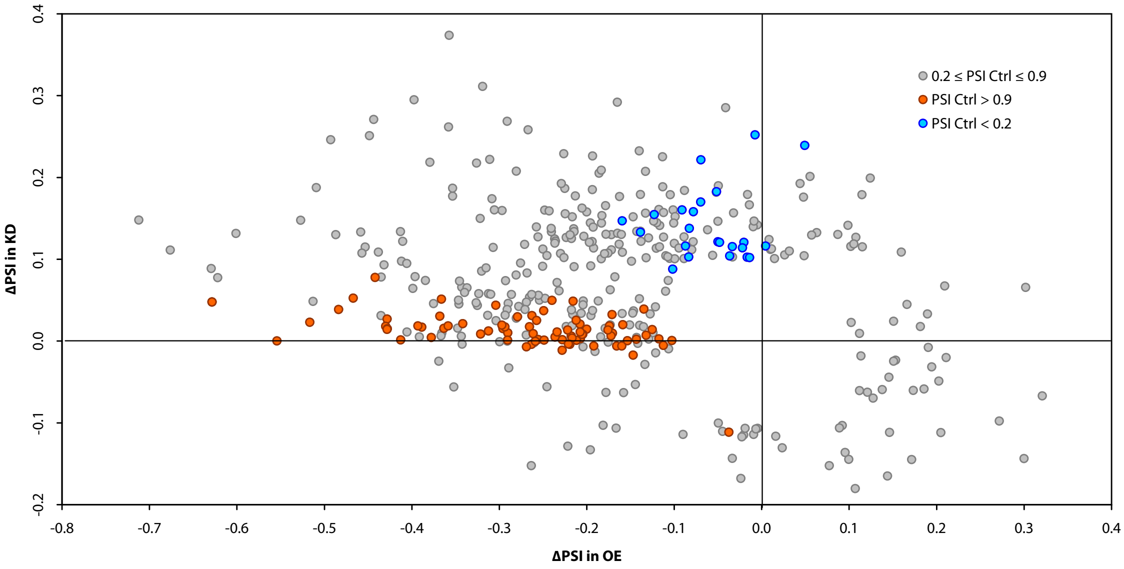

To demonstrate such changes were not false positive, using qPCR, we validated the splicing changes of four exons, which are positively correlated between OE and KD (see Fig 3A-B and supplementary Fig. 3).

The authors report 17 events that validated via qPCR - however, it is difficult to determine from the data shown how representative these events are, as 11 of the 17 have >~2-fold changes in PSO whereas the majority of events in figure 3a have deltaPSI <|0.2|. Additionally, detecting splicing changes by qPCR requires a fair amount of normalization calculations, but these validations can be done more simply with RT-PCR and running agarose gels. Can the splicing changes be detected this way or were they only detectable by qPCR?

We now marked the 21 events that were validated by using qPCR in Fig. 3A. As shown there, these events could represent the events induced by OE and KD. As mentioned above, we now validated the splicing changes of four exons, which are positively correlated between OE and KD. To assure that the splicing changes could be detected via both qPCR and normal RT-PCR followed by running Agilent Bioanalyzer, we validated the splicing changes of 8 exons also using the latter approach. As shown in the figure below, the changes measured by both approaches correlated well.

© EMBO

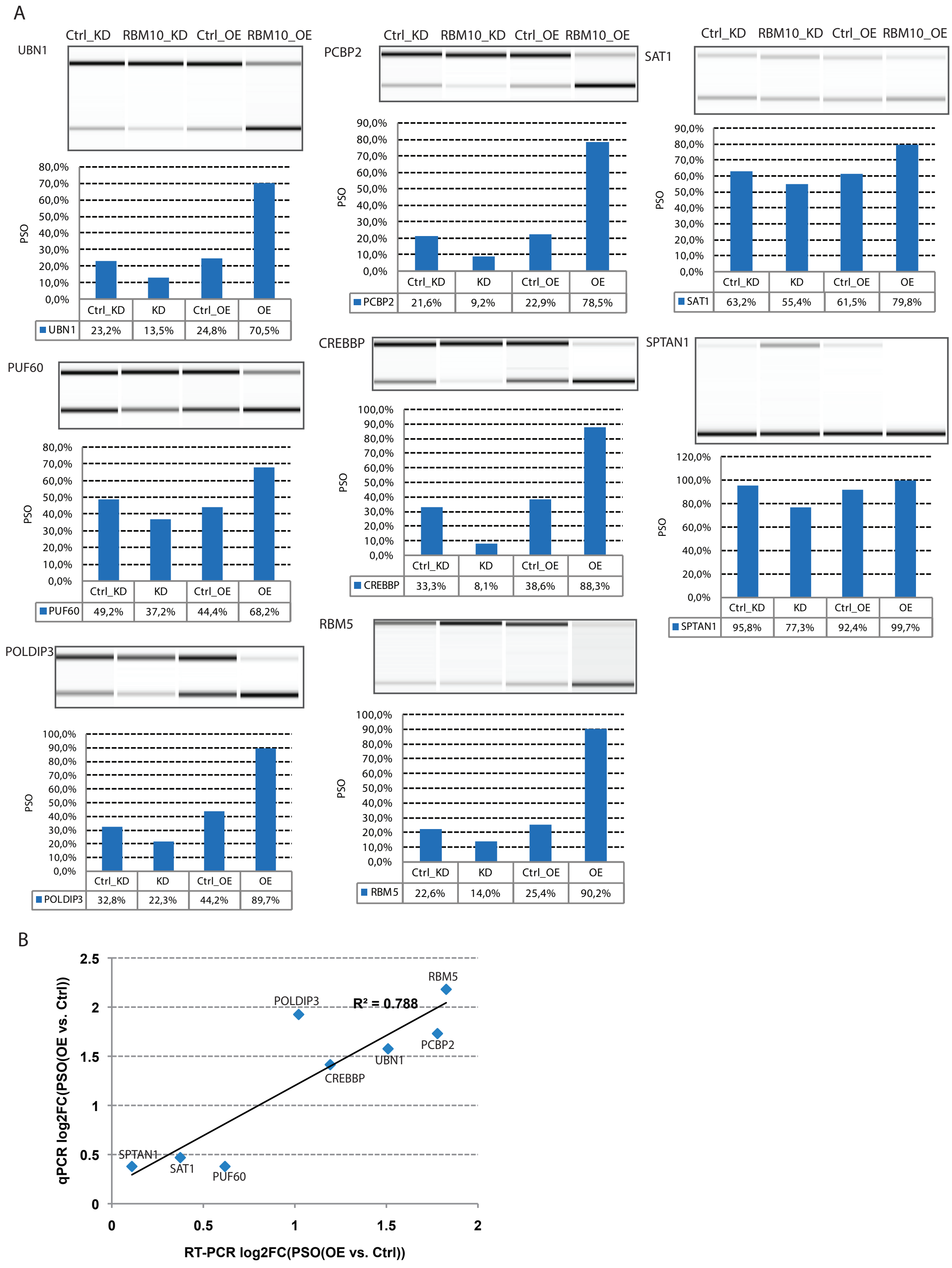

A: Up panel, Agilent Bioanalyzer gel image, Low panel: quantification of Percentage Splicing Out (PSO). B. Change in PSO measured by qPCR (x axis) correlated well with that measured by RT-PCR followed by Agilent Bioanalyzer quantification.

Figure 3c shows a track labeled "ctrl" - is this the control for the OE or the KD? (The authors list independent control datasets for both experiments - hopefully separate controls were used in all splicing experiments throughout the paper. This should be made explicit and corrected if not).

We performed separate control experiments for all the KD and OE, see supplementary table 2. In all analysis steps, we always compared each data set to its corresponding control set. In Figure 3C, the tracks for OE Ctrl and KD Ctrl looked very similar so, for simplicity, we showed only one track combining all control experiments.

Splicing maps are made with the RBM10 OE data, what do they look like with the KD data - hopefully the same.

We now included a RBM10 splicing map using KD data as supp fig 4. As shown there, the effect of RBM10 intronic binding in the vicinity of splicing sites is similar, but subtler, since in general, OE induced stronger effects than KD (see also Fig. 3A).

What fraction of the events that change are associated with RBM10 binding sites? Conversely, the PAR-CLIP experiment identified many (~88k) binding sites - what percent of binding sites are associated with regulated targets?

As explained above, we did not find clear correlation between exonic binding and splicing changes upon RBM10 perturbation. Therefore, we focused only on the intronic binding sites close to splicing sites. In total, there are 20,503 binding sites were within 150nt from 5' or 3' splicing sites. There are 5262 non-constitutive cassette exons associated with at least one such RBM10 binding sites within adjacent introns.

On one hand, whereas 982 (4.3%) and 2244 (9.9%) exons without such RBM10 binding sites showed splicing change at the level of Z<=-2 and Z<=-1, 455 (8%) and 878 (16.7%), exons with binding at >= one splicing sites showed splicing change at the level of Z<=-2 and Z<=-1. Therefore, it is clear that exons with RBM10 binding showed stronger skipping upon RBM10 OE. However, given the complicate splicing regulatory network, it is conceivable not all the binding events could lead to significant splicing changes. The similar phenomenon is often observed in the study of transcription factors.

On the other hand, there are in total 1477 and 3122 exons with splicing change at the level of Z<=-2 and Z<=-1, respectively, of which 455 (30.8%) and 878 (28.1%) were associated with at least one RBM10 binding at the splicing sites of adjacent introns. The remaining exons without such RBM10 binding sites could well represent the secondary effect given that a number of known splicing regulators were differentially expressed/spliced upon RBM10 perturbation.

Finally, we detected in total 412 exons with significant splicing changes (fdr < 0.05, |ΔPSI| ≥ 10%), of which 127 (30.8%) were associated with at least one RBM10 binding at the splicing sites of adjacent introns. Again, the remaining ones could well represent the secondary effect.

RBM10 splicing models:
The minigene experiments seem like a nice idea, but I unfortunately I don't think the results are as convincing as claimed. In Figure 4, the authors state that "These data provide unequivocal support to our hypothesis that RBM10 binding ... would facilitate the skipping of cassette exons", but the lack of technical qPCR error bars as well as some sort of statistical test to show whether the observed changes are significant conflict with such a strong statement.

In Fig. 4B and C, we showed the results from the three replicates. Using two tailed paired t test, comparing the RBM10 induced splicing changes between the minigenes containing wild-type or mutant RBM10-binding sites, the observed differences (except Del_U3 for *PUF60*), although subtle in absolute values, are indeed statistically significant. We now added the p-value to the legend of Fig. 4B and C.

Additionally, the effect on splicing for three of the four mutations made in Fig 4b/c is extremely small (~5% or less), and the data shown only reflects the change in exon exclusion between OE and control. I was curious as to whether the mutations performed show significantly altered PSO rates in the control (or over-expression) by themselves, and to what the rates of exon inclusion for these

events typically are (i.e., if they were 90% excluded in the control then a small effect upon RBM10 OE would be not surprising).

Since the binding sites are close to the splicing site, unsurprisingly, once mutated, the splicing pattern even in the control was changed (see the supp Fig. 5). The PSO rates in the minigene without RBM10 OE ranged from 30% to 90%.

Additionally, it would be ideal to perform the direct experiment (forced RBM10 association with intronic loci, e.g. by MS2 tagging) to prove that RBM10 recruitment to an intron will alter splice site choice.

We thank the referee for the suggestion. To further test the effect of RBM10 intronic binding on exon skipping, we performed two additional minigene experiments in a heterologous context.

First, we inserted RBM10 binding sites into intronic location of a new splicing reporter, pZW2C, which was constructed by inserting exon 2 of the Chinese hamster dihydrofolate reductase gene and part of its flanking introns between two GFP exons (Figure 4D) (Wang et al, 2013). We inserted three RBM10 binding sites from *PUF60* and assayed the exon skipping changes upon RBM10 OE respectively. As shown in Figure 4E, insertion of all the three RBM10 binding sites exhibited significantly stronger skipping effects upon RBM10 OE compared with the control, demonstrating that intronic binding of RBM10 near splice sites indeed could enhance exon skipping in a heterologous context.

Second, as the referee suggested, we fused RBM10 with a modified pumilio domain, PUF3-2, which specifically recognizes an 8-nt sequence 'UGUAUGUA' with high affinity (Figure 4F) (Wang et al, 2009). Thereby, we could tether RBM10 close to (18nt downstream) splicing sites of the cassette exon (Figure 4F) (Wang et al, 2009). As shown in Figure 4G, the expression of RBM10-PUF induced strong exon skipping effects, while expression of PUF3-2 alone showed hardly any changes.

We believed the three minigene experiments together now could provide unequivocal support to our hypothesis that RBM10 binding in the vicinity of splicing sites would facilitate the skipping of cassette exons.

P-values are missing for Fig. 5d. The strength of splicing sites distal to the cassette exons does not seem significantly stronger than that of those immediately flanking the exons, and in general the effect size here seems to be very slight.

We agree that the effect looks quite slight, however, the strengths of distal 5' (3') splicing sites is significantly higher than the strengths of 5' (3', respectively) splicing sites directly flanking non-constitutive cassette exons (KS-test, $p \approx 0$). Furthermore, in Fig. 5C, (previously Fig. 5D), we want to stress that the difference is also statistically significant between the splicing sites immediately flanking all cassette exons and those with higher exclusion upon RBM10 OE. We added these p-values to the figure.

It looks like fig 6b should be recreated by the authors, seems like it was taken from another publication.

Fig. 6B was indeed created by Yongbo Wang based on Uniprot and Pfam protein domain annotation using a software called "DOG1.0" (http://dog.biocuckoo.org/). Nevertheless, to avoid any possible misunderstanding, we replaced with a new figure.

TARP syndrome analysis:
Intriguingly, the splicing changes observed here correlated well with changes induced by RBM10 KD in HEK293 (Fig. 6c). What about compared to the OE experiment?

Both RBM10 KD and the mutant RBM10 identified in the patient represent loss-of-function events. Therefore we compared in the manuscript only between the patient and RBM10 KD. As shown in

the figure below, the changes identified in the patient were inversely correlated with OE. But, as expected, the correlation coefficient is much lower in this case (R^2: 0.177 for KD *vs* 0.0252 for OE).

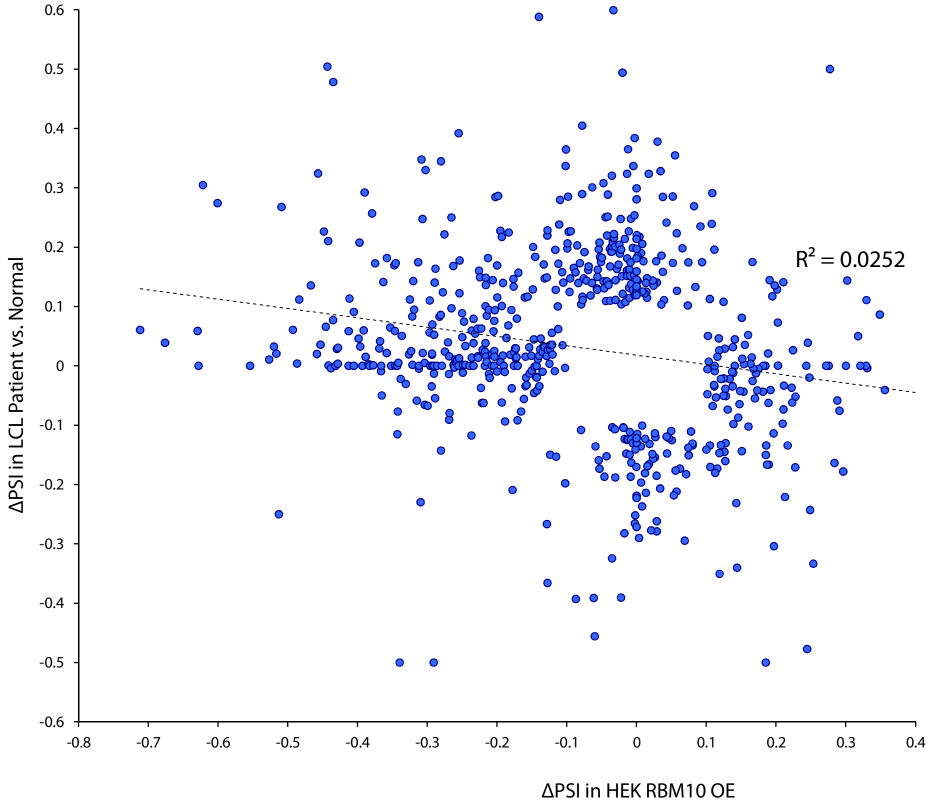

Supplemental figure 3 needs better quantification - it is clear from the figure that the mutated RBM10 is not silenced, but it is difficult to tell whether the expression is actually unchanged from the results shown.

We quantified the expression difference based on the signal intensity of the band on western blot and now included the number in the supp fig 6.

Figure 6c also needs statistics - what is the correlation between the two samples?

We included now the correlation coefficient for Figure 7C (previously Figure 6C).

Figure 6c-e are used to propose that this patient deletion acts as an RBM10 deletion through loss of nuclear expression. However, the deletion includes not only the NLS but also additional sequences. Figure 6e would be strengthened by mutating either only the NLS, or attaching a normal NLS to the mutated RBM10 to show that the molecular phenotypes observed in this patient are characteristic mis-localization of RBM10 and not also loss of function through deletion of additional domains.

The deletion removed also other functional domains, including a zinc finger domain and part of a G patch domain. However, as shown in Figure 7B, the loss of NLS will abolish the nuclear function regardless of the effect on other domains. In this manuscript, we focused on the splicing regulation mediated by RBM10 and clearly showed that the mutant identified in the patient lost such function due to mis-localization, and the splicing changes correlated well with the changes upon RBM10 KD.

Discussion comments:
Overall, the discussion needs to be rewritten to put the results of the paper in context. The idea the RBM10 works with other RBPs needs to be presented sooner than the discussion, otherwise the results section makes no sense in this context - for example, it is not until the discussion that the authors imply that RBM10 does not seem to bind the identified motifs by itself.

As the referee suggested, we moved the discussion of RBM10 exonic binding into the result part.

Similarly, the TARP section needs a better description of how it fits with the results in the rest of the paper - if RBM10 loss of function causing TARP is previously known, is the novelty the validation of the individual deletion patient as actually losing nuclear localization? Or that the loss of nuclear localization of RBM10 resembles RBM10 knockdown?

In previous findings, only nonsense or frameshift mutations in RBM10 were identified in the TARP patient. We for the first time identified an in-frame deletion and showed showed the loss of nuclear localization of RBM10 could lead to a similar phenotype, thereby demonstrating that RBM10 exerts a critical function in the cell nucleus during development. At molecular level, we also showed that mis-localized RMB10 mutant protein renders similar splicing de-regulation as RBM10 KD, supporting again that RBM10 predominantly functions in the nucleus as a novel splicing regulator.

The statement "Whole-mount in situ expression analysis of the murine Rbm10 has shown that the gene was expressed during embryonic development in a pattern consistent with the human malformations observed in TARP syndrome" is missing a reference.

We added now the reference.

The discussion refers to two experiments that need to be incorporated into the main text, as they are not mentioned anywhere in the manuscript before the discussion - one experiment (an in vitro binding RBM10 experiment) that is not shown in any figures and only mentioned in the supplemental methods, and discussion of the effect of exonic RBM10 binding (figure 7), which is also not presented in the results.

As the referee suggested, we included the gel shift assay in the supplementary figure 1 and effect of exonic RBM10 binding in the result part.

Minor comments:

-Breitling et al 2004 citation in the methods section is misformatted and not included in reference list

Thanks for pointing this out, we corrected it.

-typo in figure reference: "In agreement with our in vivo data, the skipping of the cassette exons was enhanced upon RBM10 OE (Fig. 54b)"

Thanks for pointing this out, we corrected it.

-Figure 5 figure legend is mislabeled as figure 4

Thanks for pointing this out, we corrected it.

Referee #3 (Comments on Novelty/Model System):

Technical quality
Major issues:
1. Filtering out potential contaminants. While most of the reads are derived from cross-linked RNA species (carry U to C transitions , from supplementary figure 1a it is clear that there is successful RNA pull-down from the control samples that were not cross-linked. Such result indicates that the washing stringency may not have been sufficient to remove species of RNA retained non-specifically on the resin or bound through mediated interaction. Reads derived from such non-specifically interacting RNAs can clearly be identified in PAR-CLIP by the absence of U to C transition in the sequence. Were those reads removed from the set that was used to define the RBM10 binding site?

Not all the Us in all RNAs could be labeled with 4sU, not all proteins interacted i4sUs could be crosslinked and not all crosslinked 4sUs will be converted to C during RT-PCR. Therefore, to achieve an optimal balance between sensitivity and specificity, the in-house PAR-CLIP data

analysis pipeline [also used in Lebedeva et.al. 2011] used also the reads without T to C transition, but required each cluster to contain at least one read with T to C transition. As a brief description of the pipeline, reads were firstly quality trimmed, adapters were removed. Pre-processsed reads were then mapped to the human reference genome UCSC hg19 without seeding, allowing for at most one mismatch, insertion or deletion (edit distance of 1). Clusters were called on the set of uniquely mappable reads if 1) at least two reads support the cluster and 2) at least one T->C conversion was detected within the clustered reads. The preferred cross-linked site for each cluster was defined as the site with the highest number of T->C conversion events. Clusters were scored by the number of T->C conversions. A false-discovery rate of <= 5% was obtained by filtering clusters on the quality scores, considering reads mapping antisense to annotations and reads mapping to chrY as false-positives. As shown in the figure below, the no. of PAR-CLIP reads and the reads containing T-C conversions within each cluster were highly correlated.

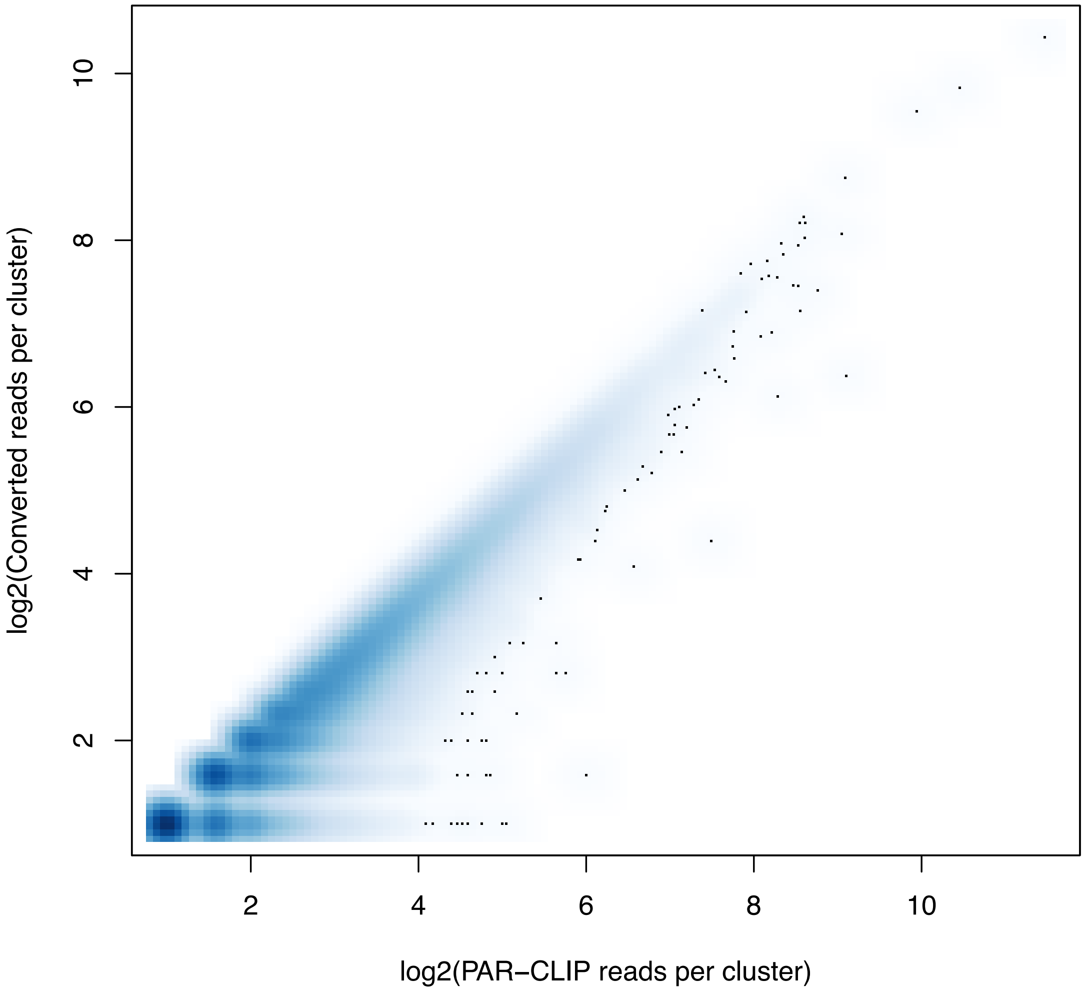

2. Binding site clusters. The authors need to provide more information on the cluster that were identified in the PAR-CLIP experiment:
(i) What is the distribution of the cluster sizes?

We included now the distribution of cluster sizes in supp figure 1. As shown there, most were between 20 and 40 nt.

(ii) How many reads are forming a typical cluster and what is the read count distribution of the clusters? The relatively small overlap between the two experiments would indicate that most of the clusters are formed by relatively few reads.

We now included the distribution of PAR-CLIP read counts for all cluster or the consensus clusters in supp figure 1. As expected, it is clear the consensus ones were formed with more PAR-CLIP

© EMBO

reads than non-overlapping ones, demonstrating the consensus binding clusters represent more stable and more likely functionally relevant events.

(iii*) Within each cluster, are there preferred cross-linking sites? This would be a strong indication of the presence of high affinity binding site.*

There are preferred cross-linking sites within each cluster. See the response above for the brief description of PAR-CLIP data analysis.

3. RBM10 binding site sequence. The authors identify GAAGA as a pentamer that is enriched in the RBM10 binding site clusters that are located within exons. The absence of Uridine residue from the binding site is quite surprising considering that PAR-CLIP is reliant on cross-linking to U residues. It raises the possibility that the approach used to identify the binding site sequence is inadequate.

In particular what was the rational to analyze separately the exonic and intronic binding sites? Do the authors expect RBM10 to bind to a different sequence in the introns compared to the exons? Was the GAAGA pentamer significantly enriched in the intronic binding sites? If not, than what is the evidence of sequence specific binding of RBM10 to RNA? Alternatively it is possible that the sequence recognized by RBM10 does not contain uridines that can be cross-linked to the protein. This would mean that PAR-CLIP is not an adequate experimental approach for identifying RBM10 binding sites and the standard CLIP approach, that uses short wavelength UV light to crosslink to unmodified RNA should be used instead.

We agree with the reviewer's view that it seems counter-intuitive to find a U depleted pentamer motif using 4-thiourdine enhanced crosslinking. However in this respect we would like to point out that there is no need for the RNA-recognition element itself to be crosslinked to the protein of interest to capture the RNA target fragment by PAR-CLIP. Hafner and colleagues showed, in their original PAR-CLIP study, that RNA-target sites of the RNA binding protein QUAKING, despite the presence of U in the recognition element, are crosslinked through uridines outside, but close proximity, of the A(C/U)UAA(C/U) recognition element (Hafner et al. Cell 2012 Figure 3E+F).

We did not expect RBM10 binding differently between exons and intron. Indeed, we started the motif analysis without separating exonic and intronic binding clusters. GAAGA and its close derivatives turned out to the only enriched motifs, and the enrichment was accounted for totally by the exonic binding clusters and there is no such enrichment in the intronic binding clusters (see also the response to referee #2). As described in the manuscript, we could not demonstrate the in vitro binding of immuno-purified RBM10 with an oligoribonucleotide containing the GAAGA motif by gel-shift assay although crude HEK 293 cell lysates did bind with the same oligoribonucleotide under the same experimental condition. This suggested this motif could not represent the specific sequences recognized by RBM10. Instead, GAAGA motif is a known binding motif of several serine/arginine-rich (SR) proteins (Long & Caceres, 2009; Sanford et al, 2009). Therefore, it is tempting to speculate that RBM10 was associated with this motif indirectly via other protein partners.

4. Binding to U2. The finding that RBM10 binds to U2 snRNA is quite intriguing. How many reads were mapped to U2 compared to the reads mapped in protein coding genes. Considering the high abundance of the snRNP RNAs, is there enrichment of RBM10 cross-links to U2 that is statistically significant?

In total, there are 0.25 million RBM10 PAR-CLIP reads mapped to U2, whereas there are 13,51 million PAR-CLIP reads mapped (including both uniquely and non-uniquely mapped reads) to all protein coding genes. However, without precise abundance estimation of U2 versus all protein coding genes, it is difficult to compare the binding of RBM10 on U2 to that on the sum of all protein coding genes. Instead, in order to estimate the statistical significance of RBM10-U2 interaction, we compared the binding of RBM10 on U2 with that of AGO2, in which the PAR-CLIP experiment was performed with the same protocol and in the same cell line. In AGO2 PAR-CLIP, only 582 PAR-CLIP reads mapped to U2 and 0.69 million PAR-CLIP reads mapped to all protein coding

genes. Using Fisher test, we can demonstrate the binding of RBM10 on U2 is statistically significant (P value < 2.2 e-16)

5. Correlation between RBM10 binding and effect on splicing. One strong aspect of the presented work is the availability of both RNA binding (PAR-CLIP) and exon inclusion data (RNA-seq). Surprisingly the authors do not provide information as to what fraction of the RBM10 regulated exons contain RBM10 binding sites. Good correlation between RBM10 binding and exon inclusion/skipping would strongly argue that the effect on splicing is specific to RBM10 and is not a secondary effect.

In total, there are 20,503 binding sites were within 150nt from 5' or 3' splicing sites. There are 5,262 non-constitutive cassette exons associated with at least one such RBM10 binding sites within adjacent introns. We detected in total 412 exons with significant splicing changes (fdr < 0.05, $|\Delta PSI| \geq 10\%$), of which 127 (30.8%) were associated with RBM10 binding on at least one splicing sites in the adjacent introns. The remaining ones could well represent the secondary effect.

See also the more extensive response to referee #2.

6. RNA splicing map. Why are the exonic sequences ignored in the RNA map (figure 3d)? This makes no sense considering that most of the RBM10 binding clusters are located in exons (figure 2b).

As described in the manuscript, we observed that RBM10 binding at exonic GAAGA sites appeared to override the exon skipping effect of intronic binding. Except this, we tried, but failed to find any additional clear correlation between exonic binding and splicing changes induced by RBM10 perturbations. Comparing with the neighboring constitutive exons, the binding is lower in all the cassette exons, the exons more excluded upon RBM10 OE, as well as the exons more included upon RBM10 OE. The fact that there is no clear difference in exon binding between different groups of exons indicates that the exon binding alone might not contribute much to splicing regulation. Having said these, we could not exclude the effect of exonic binding on other processes such as mRNA translation, which is beyond the scope of this study. See also the response to referee #2.

7. Minigene experiments (figure 4). There are multiple issues with the data presented on this figure: (i) Disrupting the binding sites does significantly disrupt the effect of RBM10 on splicing, possible with the exception of Mut D5. This is a very strong argument that the authors did not identify the correct binding sites. This data not only does not "provide unequivocal support", but directly contradicts the authors conclusion that "...RBM10 binding in the vicinity of splice sites of flanking introns would facilitate the skipping of the cassette exons".

Given that a number of genes differentially expressed/spliced upon RBM10 OE, it is conceivable that the fact that disrupting the binding sites does not totally abolish the effect of RBM10 on splicing is probably due to the secondary effects, which do not depend on direct RBM10-RNA interaction. Therefore to further prove our hypothesis, we performed two additional minigene experiments in a heterologous context, as the referee suggested. See also the response to referee #2

(ii) While the RBM10 effect on PUF60 splicing is mostly abolished in PUF60 Mut D5, this is by no means conclusive evidence that the site disrupted in Mut D5 is the cis-acting element that is recognized by RBM10. The mutation may have disrupted sequence element recognized by a different protein that is required for regulation of the alternative exon. The authors need to show that placing the RBM10 binding sites in vicinity of a heterogonous alternative exon confers regulation by RBM10.

To validate the impact of RBM10 binding on alternative splicing, we performed two additional minigene experiments in a heterologous context. See the more extensive response to referee #2.

(iii) There is no statistical analysis to show how significant the changes in exon inclusion are and what is the variability of the assay.

In Fig. 4B and C, we showed the results from the three replicates. Using two tailed paired t test, comparing the RBM10 induced splicing changes between wild type minigene and minigene containing mutant binding sites, the observed differences (except PUF60 Del_U3), although subtle in absolute values, are indeed statistically significant. We now added the p value to the legend of Fig. 4B and C.

(iv) The authors need to show the sequences of the wild type and mutated binding sites.

We listed the sequences now in Supp table 6.

(v) the Authors need to show gel images of the RT-PCR reactions.

We showed now both agarose and Agilent bioanalyzer gel images in Supp fig 5 . Given the very subtle changes between different conditions, we used the Agilent bioanalyzer for quantification, which is much more sensitive and accurate.

8. *The authors convincingly show the effect of RBM10 mutations in patients on alternative splicing. However RNA binding proteins frequently multitask and regulate RNA stability and translation. It would be interesting to know if there are transcripts with altered abundance and the patient lymphoblasts and after the RBM10 knockdown in HEK cells.*

We now added a paragraph describing the gene expression changes induced by RBM10 OE/KD in the result part and add one supplementary table listing all the genes differentially expressed upon RBM10 perturbation. Notably, the expression changes induced by KD and OE were not inversely correlated, indicating these events might not be directly regulated by RBM10. See also the response to referee #2.

Also does RBM10 bind to the mRNA UTRs, which are frequently involved in regulation of translation and RNA stability. The answers to these questions should already be in the PAR-CLIP and RNA-Seq data. The fact that the two patients presented in this study display milder phenotypes compared to the typical TARP syndrome, despite having impaired function of RBM10 in slicing would argue that the protein may have additional functions in the cytoplasm.

RBM10 binds both 5' and 3' UTR (see Figure 2). However, comparing with coding sequences, the binding at the UTR regions is much less enriched (see also the response to referee #2.). We tried to associate the UTR binding to the change of gene expression induced by RBM10 OE or KD, but could not observe any clear correlation. Having said that, again we could not exclude the possible effect of UTR binding, such as translational control. We fully agree with the referee, given the milder phenotype presented in our patients, it is conceivable that the mutant RBM10 might retain additional functions in the cytoplasm. To address this awaits future study.

Minor issues:
1. Figure 1b. From the methods it is unclear why the values for the consensus clusters are higher than the values for the reads. If clusters are aggregates of reads, one would expect the cluster density to be less than the read density.

We apologize for causing such confusion. In the Y axis, it is the density of reads and binding clusters that are represented, which are normalized by the total no. of reads or clusters.

2. On Figure 2c the authors need to define what is considered to be "Strong", "Medium" and "Weak" binding site.

We defined in the revised method part. See also the response to referee #2.

3. On Figure 3c showing the aligned PAR-CLIP reads, rather than a triangle will be more informative to the reader.

In limited space, showing all the aligned PAR-CLIP reads would make the figure quite messy. Upon the acceptance of the manuscript, we will upload the PAR-CLIP dataset into the database server DORINA hosted in our institute (http://dorina/rbp_browser/dorina.html). In DORINA, all the

aligned reads could be displayed in an interactive manner in UCSC genome browser. We believe this would be the best solution for visualizing our PAR-CLIP data.

Novelty
Although association with the spliceosome raises the possibility that RBM10 regulates alternative splicing this has not been shown to date. Furthermore, the rich sequence data obtained in this study can provide significant insight into the mechanisms by which RBM10 regulates splicing.

We thank the referee for his/her appreciation of the novelty of our study.

Medical Impact
Mutations in RBM10 have been associated with developmental disorders. Furthermore, it is frequently mutated in certain types of cancer. Understanding its function may contribute to developing cancer therapies and prognostic markers.

We thank the referee for his/her appreciation of the relevance of our study.

Adequacy of the model system
The authors express epitope tagged RBM10 for the PAR-CLIP experiments. While this is acceptable, particularly in cases where good quality antibodies are not immediately available for the endogenous protein, the authors need to show that the levels of the expressed protein are comparable to those of the endogenous protein. Maintaining physiological protein levels is critical as over-expression may result in binding to low affinity sites on the RNA that are not occupied under normal conditions.

We are aware of the possible irrelevant effects induced by uncontrolled protein overexpression. Therefore, before performing PAR-CLIP as well as RNA-Seq, we measured the expression level of RBM10 under a series of concentration of doxycyline (DOX) and chose the one with the lowest induction level, i.e. approximately 2.5 fold overexpression comparing with endogenous expression level, induced by 10 ng/mL DOX (see Supp Fig. 2B)

Referee #3 (Remarks):

RBM10 is an RNA binding protein that has been associated in several studies with the spliceosome. However, its function there has remained unclear. The work presented by Wang et al in this manuscript ascribes a function of RBM10 in splicing and more specifically in exon recognition. The authors use a combination of PAR-CLIP and RNA-seq to build an integrated model for RNA splicing regulation by RBM10. Wang et al also show that alternative splicing patterns in patients with TARP syndrome resemble those of RBM10 knockout cell lines, concluding that the splicing regulatory function of RBM10 is disrupted in the patients. The results of the presented work can potentially have a significant impact on our understanding of splicing regulation in organism development and human disorders including cancer. While the quality of the raw data appears to be adequate the subsequent analysis leaves a lot to be desired (see the specific comments for details). The results as presented in the manuscript do not support the proposed RBM10 binding site sequence and the model for splicing regulation by RBM10. There are also some issues that need to be addressed in respect to the model system. In particular, the use of protein over-expression in the PAR-CLIP experiments may result in the identification of binding sites that are not normally occupied by RBM10. If these deficiencies are adequately addressed the work by Wang et al will without doubt have significant impact.

We thank the referee for his/her appreciation of the novelty and relevance of our study, and thank him/her for his/her critical comments. Following his/her suggestions, we carried out further computational and experimental work. We believe that our additional results substantially improved the quality of the paper and addressed the requests made by the referee.

2nd Editorial Decision 04 July 2013

Thank you for the submission of your revised manuscript to EMBO Molecular Medicine.

We have now heard back from the two Reviewers whom we asked to re-evaluate your revised manuscript.
You will see that two out of three Reviewers have remaining issues that prevent us from considering publication at this time.

Reviewer 2 notes that the finding that RBM10 binds to U2snRNA opens up to different interpretations of your data. S/he also challenges the conclusion that RBM10 binds GAAGA and would like to see stronger statistical support that correct nucleotide controls were considered. This Reviewer also notes a certain ambivalence with respect to the results on exonic binding of RBM10. Finally, Reviewer2 disagrees that the splicing changes from the patient correlate well with RBM10 knock down in 293T cells and would like you to verify how specific the correlation is by checking other large-scale datasets.

Reviewer 3 has some remaining concerns as well and is more blunt concerning the data on the RBM10 binding site and the CLIP data and suggests that they be removed altogether, while moving supplementary figures S2 and 3 to the main body. Since Reviewer 2 also has reservations on the CLIP data I would suggest that you comply with Reviewer 3's request on the CLIP data and include the RBM10 binding site data only if you can provide further experimental support as indicated by Reviewer 2.

As you know, we would normally not allow a second revision. However, after consulting with an external expert, I am prepared in this case, to give you another opportunity to improve your manuscript, with the understanding that the Reviewers' concerns must be fully addressed with additional experimental data where appropriate. Please also provide an additional copy of the revised manuscript with the changes highlighted in colour.

If you fully comply with the requested changes, the final decision will be possibly made at the Editorial level.

I look forward to seeing a revised form of your manuscript as soon as possible.

2nd Revision 06 July 2013

I would like to thank you for giving us another opportunity to improve our manuscript. After reading carefully your suggestions and the remaining concerns raised by both referees, we would like to make the changes as described below.

We feel that the complete removal of PAR-CLIP datasets, as recommened by referee 3, is not appropriate because 1) the strength of our study is that we identified not only splicing changes induced by RBM10 perturbation but also transcriptome-wide RBM10 binding sites. Only after the integrative analysis of both datasets we could reveal the molecular mechanisms underlying RBM10 mediated splicing regulation. Removal of the whole PAR-CLIP result will make the manuscript incomprehsible. 2) Our PAR-CLIP result is solid. As shown in FigS1, we have extensively demonstrated the quality by different metrics as well as the biological replicates, which showed similar performance as other published PAR-CLIP datasets (e.g. Hafner M, Cell 2010; Lebedeva S, Molecular Cell 2011). Especailly, the RBM10 intronic binding could be associated with RBM10 mediated splicing changes and the resulting mechanistic model could be unambiguously validated with minigene experiments.

As suggested by you and referee 2, we planned to take some unessential parts out or move into supporting information, which includes,

1) We removed Fig.1.

2) We removed ‘Sequence features associated with RBM10 binding’, although we think the enrichment of GAAGA motifs close to exonic RBM10 binding sites could not be explained by any nucleotide bias in exons, as claimed by referee 2. As described in Method section “Analysis of sequence features around RBM10 binding sites”, we generated an appropriate background model for motif analysis and control sites were picked only from exons when defining exonic binding motifs. We also avoided any possible bias due to exon length or distances between binding sites and exon boundaries.

3) We moved the ‚RBM10 binds to U2 snRNA’ into Supporting information, as suggested by referee 2.

4) We feel the three minigene experiments are redundant and now left only the last one with RBM10-PUF fusion experiment.

5) We removed the second mechanistic model with the effect of RBM10 exonic binding, which has not been substantiated by independent validation experiments.

Finally, as to the correlation between splicing changes from the patient and those induced by RBM10 knockdown in 293T cells, it is significant ($p<10e-16$) and given that they were measured in different cell types, a higher correlation coefficient should not be expected. Therefore we do not believe it is just some noise or stochastic effects and the comparison with the data from other splicing factor knockdown in 293 cells is not necessary.

I hope you would find the changes listed above acceptable. I am looking forward to your editorial decision.

© EMBO
