# Supplementary material for: Integrative analysis revealed the molecular mechanism underlying RBM10-mediated splicing regulation
Source: EMBO Mol Med. 2013 Aug 22;5(9):1431–42. doi: 10.1002/emmm.201302663 (PMC3799496; doi:10.1002/emmm.201302663)
Supplement: Supplementary file 2 [file emmm0005-1431-SD2.pdf]

Supporting Information for

"Integrative analysis revealed the molecular mechanism underlying RBM10-mediated splicing regulation"

RBM10 binding to U2 snRNA

Detailed report and discussion of clinical findings

Figure S1. RBM10 PAR-CLIP experiments and results

Figure S2. RNA-seq experiments and results

Figure S3. qPCR validation of splicing changes detected by RNA-seq

Figure S4. Cumulative distribution functions of splicing changes upon RBM10 KD.

Figure S5. Representative agarose gel and bioanalyzer gel image in minigene experiments.

Figure S6. Validation of an *in-frame* deletion of *RBM10* detected in a patient with TARP syndrome

Figure S7. Photos from the two patients reported in this study

Figure S8. RBM10 binding to U2 snRNA

Table S1. Summary of PAR-CLIP sequencing results

Table S2. Summary of RNA sequencing results

Table S3. Differentially expressed genes upon RBM10 perturbation

Table S4. Differentially spliced exons upon RBM10 perturbation

Table S5. Primer sequences

Table S6. Summary of phenotypic spectrum in TARP patients and our patients

Supplementary Methods

RBM10 binding to U2 snRNA

After identifying the binding sites in the pre-mRNA targets, we examined whether RBM10 was associated with splicing snRNAs, considering that splicing regulators might associate with pre-mRNA substrates and spliceosomal snRNPs concomitantly to control the splicing outcomes (Chen & Manley, 2009; Will & Lührmann, 2010). For this purpose, we mapped PAR-CLIP reads to snRNA gene loci and calculated the reads distribution for both major and minor spliceosomal snRNAs (Materials and methods). As shown in Fig S8A, the majority of the reads were located in U2 snRNA. To examine the binding pattern of RBM10 on U2 snRNA in more detail, we plotted the number of PAR-CLIP reads containing T to C transition events along the U2 snRNA consensus sequences (Fig. S8B). RBM10 exhibited apparent crosslinking at two positions, which, interestingly, were very close to the U2 conserved sequences responsible for branching site pairing (Fig. S8C). Together with the preferential binding close to intronic splice sites, these observations indicate that RBM10 very likely involves in splice site recognition and/or pairing, as well as further intron removal processes via coordinated interactions with snRNPs and the pre-mRNA substrates.

Detailed report and discussion of clinical findings

Patient III:1 was born at 35 weeks of gestation per caesarean section with normal birth measurements [weight: 1980 g (-1 SD), length: 45 cm (-1.5 SD), OFC: 31.5 cm (-1 SD)]. He was severely hypotonic. A diaphragmatic hernia, a Dandy-Walker malformation and a ventricular septal defect were diagnosed. His psychomotor development was severely delayed: He learned sitting without support at the age of two and spoke single words, but unlearned them. He was never able to walk. Obstipation was present in early childhood.

Last clinical evaluation was performed at the age of 11 years. He suffered from myelodysplastic syndrome since three years and his general state of health was impaired. His body measurements were low [height: 119 cm (-3.9 SD), weight: 18 kg (BMI: 12), OFC: 49 cm (-3.1 SD)]. He had an absence epilepsy which was treated with levetiracetam, ethosuccimide and clobazam, an colitis ulcerosa, a primary sclerosing cholangitis and a renal insufficiency. Clinical evaluation revealed a trigonocephaly, curly hair, absence of lateral eyebrows, telecanthus with an intercanthal distance of 3.5 cm, nystagmus, large mouth with thin upper lip and small chin. He had a cleft palate and a hypoplastic uvula. His ears are small and low-set. He had contractures of both hands with ulnar deviation and pes equinus. In addition, he presented with cryptorchidism and micropenis. He deceased at the age of 14 $^{2}/_{12}$ years because of his myelodysplastic syndrome.

Patient III:2 is the maternal cousin of patient III:1. He was born at 39 weeks of gestation also with low birth measurements: weight 2140 g (-3.1 SD), 43 cm (-3.9 SD), OFC 31 cm (-3.3 SD). Again a severe muscle hypotonia were noted. He had a cerebellar vermis hypoplasia and presented with severe psychomotor delay. He also had a ventricular septal defect and developed a scoliosis. Last clinical examination was performed at the age of 10 years. His body measurements were low [height: 116 cm (-3.9 SD), weight: 15 kg (BMI: 11), OFC: 48.5 cm (-3.3 SD). He presented with curly hair, mild hypertelorism, large mouth with thin upper lip, hypoplastic uvula and contractures of hands and feet, scoliosis and cryptorchidism. He was unable to sit without support and unable to speak a single word. His general status of health was better than that of his cousin.

The main clinical findings of TARP syndrome are Talipes equinovarus, Atrial septal defect, Robin sequence, and Persistence of the left superior vena cava. Talipes and Robin sequence were present in both cousins. However, in one boy, a different congenital heart defect, ventricular septal defect, was manifested. Persistence of the left superior vena cava was not observed in both patients (Supplementary Table 7).

The two patients are the eldest patients reported so far. Thus, consistent with previous report, TARP syndrome is not always associated with pre- or perinatal death (Gripp

et.al, 2011). Similar as those patients, who survived the neonatal period, intellectual disability (ID) is severe in both patients, who were unable to speak and to walk without support. As depicted in Supplementary Figure 7, the affected boys show similar craniofacial dysmorphism: curly hair, hypertelorism, small, posterior rotated and low-set ears, a large mouth with thin upper and thick lower lip. Unfortunately, except the Mexican patient reported by Gripp et al. (Gripp et.al, 2011), photographs from the other previously published patients are not available. The description of additional patients with TARP syndrome will demonstrate whether there is a recognizable craniofacial phenotype. So far unreported clinical findings were observed in the boys reported here and allowed to widen the clinical spectrum of TARP syndrome: Dandy-Walker malformation, optic atrophy, diaphragmatic hernia, and micropenis. The index patient was also affected with colitis ulcerosa and a myelodysplastic syndrome, which might be part of the syndrome. All together, these findings clearly demonstrate that the phenotype of TARP syndrome is more variable than previously reported.

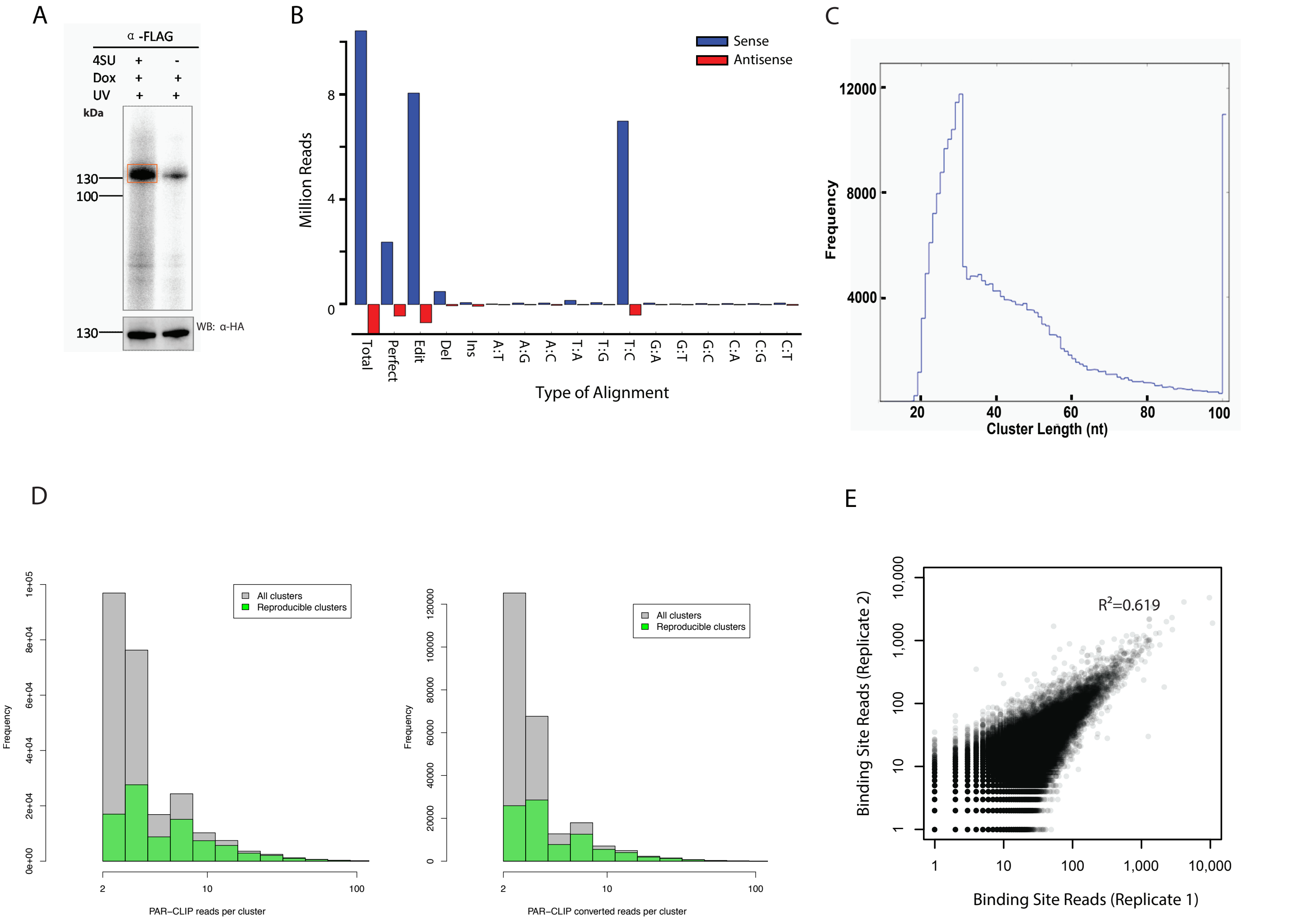

Figure S1. RBM10 PAR-CLIP experiments and results.

(A) Phosphorimages of SDS-PAGE that resolved ^{32}P-labeled RNA–FLAG/HA-RBM10 complexes immunoprecipitated (IP) from lysates of HEK293 cells cultured in media in the absence or presence of 100 µM photoactivatable 4sU and crosslinked with UV 365 nm. It was clear that RBM10 indeed bound with RNA and that the addition of 4sU greatly enhanced the crosslinking efficiency. (B) Distribution of specific mismatches in aligned PAR-CLIP reads. The predominate T to C mismatches are signature of efficient crosslinking. (C) Length distribution of RBM10 binding clusters. (D) Distribution of number of PAR-CLIP reads (left) or PAR-CLIP reads containing T-C conversions (right) within all the clusters or the consensus clusters. (E) Correlation of RBM10 binding affinities of consensus clusters measured in the two replicates (i.e the number of PAR-CLIP reads spanning the preferred crosslinking sites).

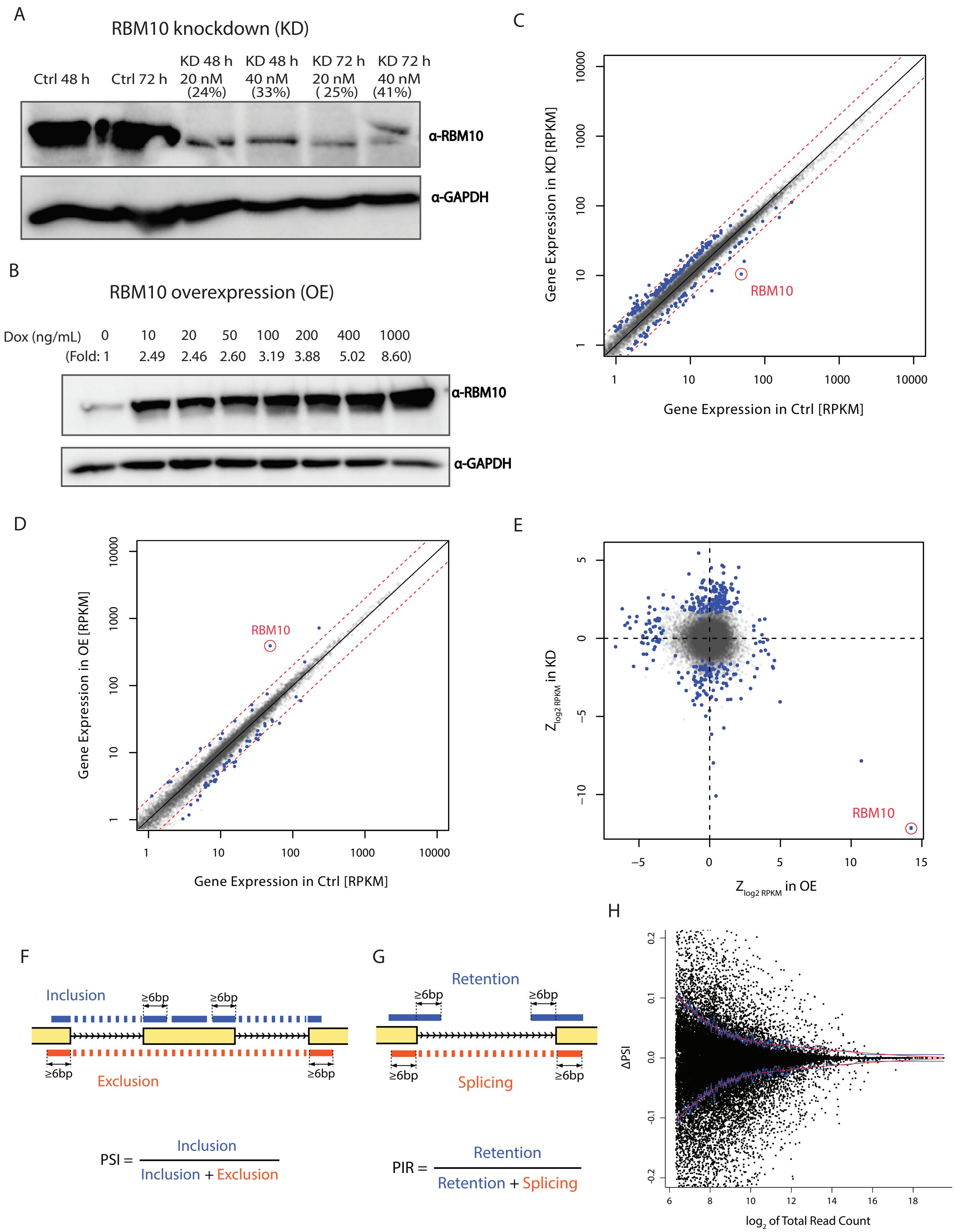
A
RBM10 knockdown (KD)
Ctrl 48 h
Ctrl 72 h
KD 48 h 20 nM (24%)
KD 48 h 40 nM (33%)
KD 72 h 20 nM (25%)
KD 72 h 40 nM (41%)
α-RBM10
α-GAPDH
B
RBM10 overexpression (OE)
Dox (ng/mL) 0 10 20 50 100 200 400 1000
(Fold: 1 2.49 2.46 2.60 3.19 3.88 5.02 8.60)
α-RBM10
α-GAPDH
C
Gene Expression in KD [RPKM]
Gene Expression in Ctrl [RPKM]
RBM10
D
Gene Expression in OE [RPKM]
Gene Expression in Ctrl [RPKM]
RBM10
E
Z log2 RPKM in KD
Z log2 RPKM in OE
RBM10
F
Inclusion
Exclusion
≥6bp
PSI = Inclusion / Inclusion + Exclusion
G
Retention
Splicing
≥6bp
PIR = Retention / Retention + Splicing
H
ΔPSI
log2 of Total Read Count

Figure S2. RNA-seq experiments and results.

(A) RBM10 knockdown (KD) efficiency. Assessment of RBM10 KD efficiency by qPCR and western blot. Compared with control, 48 and 72 hrs after KD, the RBM10 protein level was decreased to approximately 24-41%. For RNA-seq experiments, we harvested the cells 24, 48 and 72 hrs after KD, see Supporting Information Table S2. (B) RBM10 overexpression (OE) efficiency. Assessment of RBM10 OE efficiency by western blot. Compared with control, after OE, the RBM10 protein level was increased by 2-9 fold upon stimulation with different concentration of Dox. For RNA-seq and PAR-CLIP experiments, we used the cells stimulated with 10ng/mL Dox. (C) Gene expression changes after RBM10 KD. In the scatterplot, gene expression levels (in RPKM) of control cells (X axis) were plotted against that of cells after KD (Y axis). Differentially expressed genes were marked in blue. (D) Gene expression changes after RBM10 OE. In the scatterplot, gene expression levels (in RPKM) of control cells (X axis) were plotted against that of cells after OE (Y axis). Differentially expressed genes were marked in blue. (E) Gene expression changes (Z value) induced by RBM10 KD (X axis) were plotted against those induced by RBM10 OE (Y axis). Differentially expressed genes in either condition were marked in blue. (F) Scheme for computing the percent spliced-in (PSI) value of the middle exon. (G) Scheme for computing the percent intron retention (PIR) value of an intron. (H) MA plot with ΔPSI of exons between RBM10 OE and Control at y-axis and the number of all reads used for calculating the ΔPSI at X axis. The blue line denotes the local standard deviation (window size = 1% of exons), the red line denotes loess line.

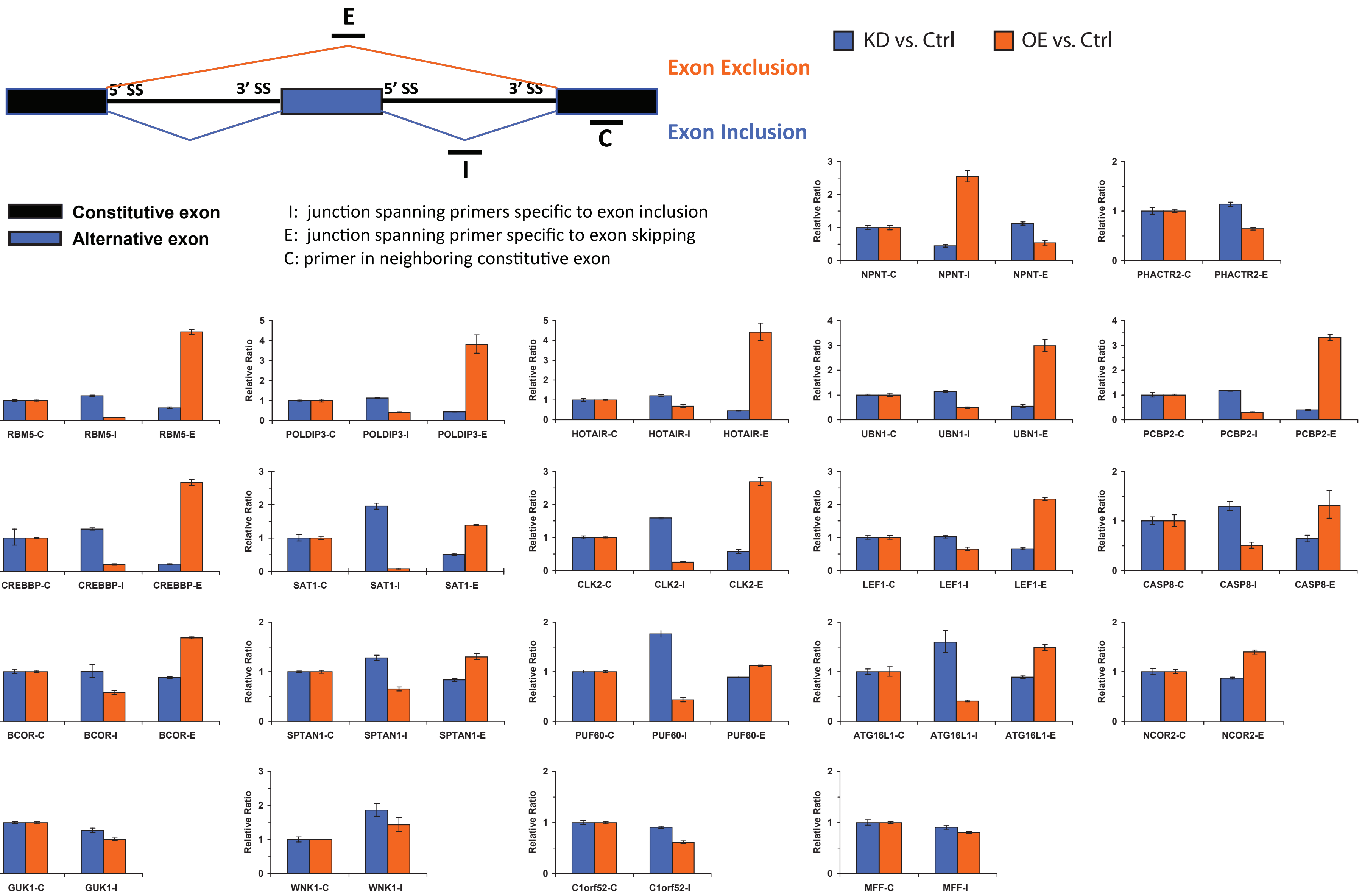
E
5' SS
3' SS
5' SS
3' SS
C
I
Exon Exclusion
Exon Inclusion
KD vs. Ctrl
OE vs. Ctrl
Constitutive exon
Alternative exon
I: junction spanning primers specific to exon inclusion
E: junction spanning primer specific to exon skipping
C: primer in neighboring constitutive exon
Relative Ratio
NPNT-C
NPNT-I
NPNT-E
PHACTR2-C
PHACTR2-E
RBM5-C
RBM5-I
RBM5-E
POLDIP3-C
POLDIP3-I
POLDIP3-E
HOTAIR-C
HOTAIR-I
HOTAIR-E
UBN1-C
UBN1-I
UBN1-E
PCBP2-C
PCBP2-I
PCBP2-E
CREBBP-C
CREBBP-I
CREBBP-E
SAT1-C
SAT1-I
SAT1-E
CLK2-C
CLK2-I
CLK2-E
LEF1-C
LEF1-I
LEF1-E
CASP8-C
CASP8-I
CASP8-E
BCOR-C
BCOR-I
BCOR-E
SPTAN1-C
SPTAN1-I
SPTAN1-E
PUF60-C
PUF60-I
PUF60-E
ATG16L1-C
ATG16L1-I
ATG16L1-E
NCOR2-C
NCOR2-E
GUK1-C
GUK1-I
WNK1-C
WNK1-I
C1orf52-C
C1orf52-I
MFF-C
MFF-I

Figure S3. qPCR validation of splicing changes detected by RNA-seq.

qPCR validation of 21 splicing changes identified based on RNA-seq data. Primers targeting transcript isoforms including (I) or excluding (E) the 21 cassette exons (Table S5) were used to measure the expression level of the respective isoforms, which was then normalized based on the expression level of the neighboring constitutive exons (C).

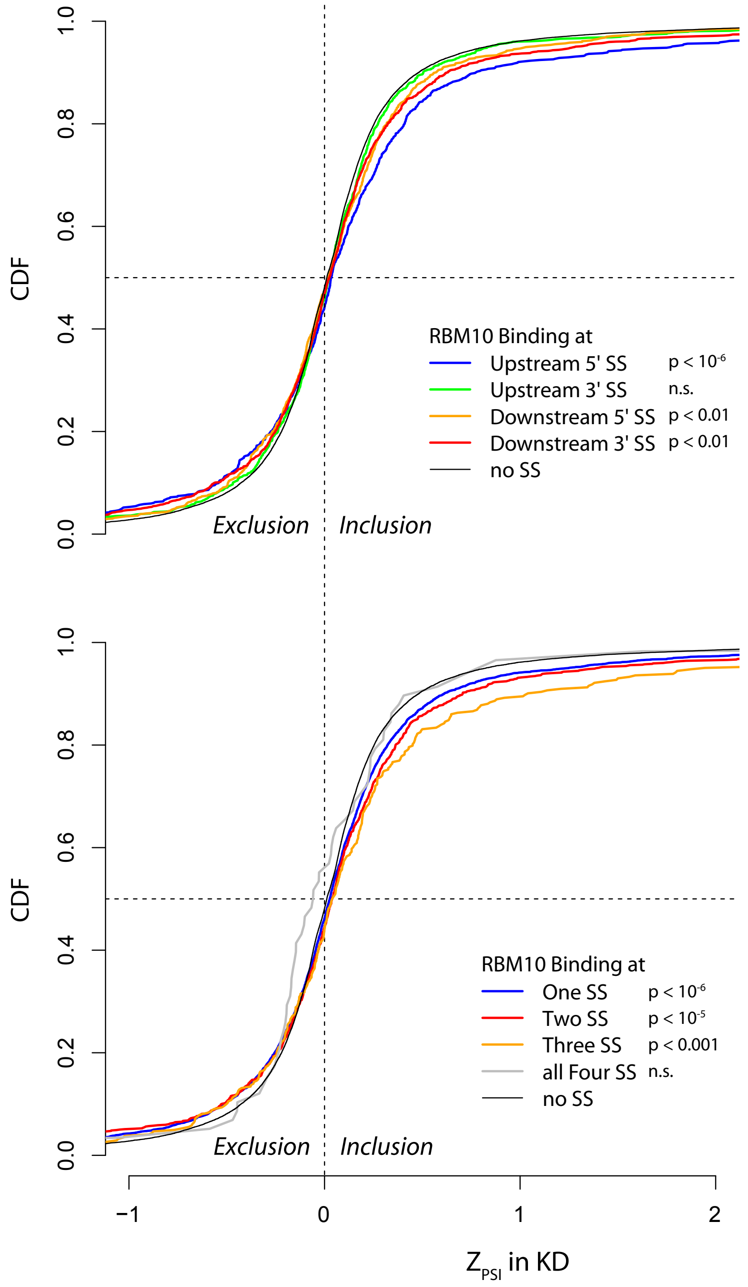

Figure S4. Cumulative distribution functions of splicing changes upon RBM10 KD. Cumulative distribution functions of splicing changes upon RBM10 KD for different groups of cassette exons with RBM10 binding close to none or one of the four splicing sites of the adjacent introns (upper panel), or to different number of the four splicing sites (Lower panel). The exons with RBM10 binding close to one of the four splice sites were more likely included upon RBM10 KD, and those with binding close to 3' splice sites of upstream introns exhibiting the weakest and insignificant inclusion propensity. Intriguingly, exons with binding close to more of the four splice sites showed progressively stronger inclusion tendency upon RBM10 KD (Please note 1) the group of exons with binding close to all four splice sites was

too small to be reliable and 2) compared with RBM10 OE induced changes, the splicing changed induced by KD was overall weaker).

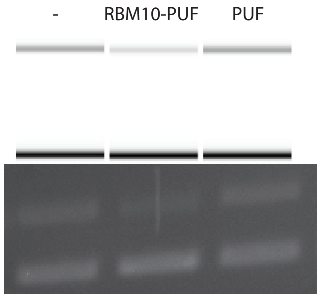

Figure S5. Representative agarose gel (lower panel) and bioanalyzer gel image (upper panel) in minigene experiments. Label is the same as Fig 4.

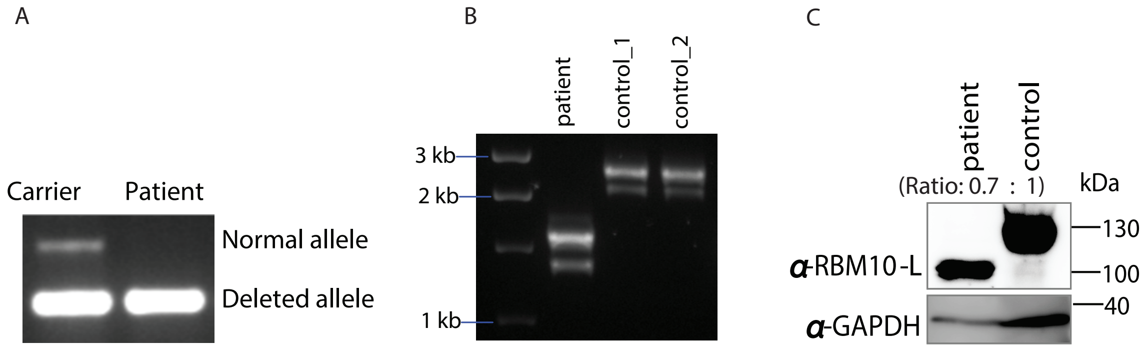

Figure S6.Validation of an *in-frame* deletion of *RBM10* detected in a patient with TARP syndrome. (A) PCR on genomic DNA from the patient (III:1) and his mother (Carrier) with a combination of two primer sets, one within the deletion and the other flanking the deletion, was used to validate the deletion. Using a single primer set that flanked the deletion failed to amplify the normal allele in heterozygous females. The primer sequences were listed in Table S5. (B) RT-PCR performed on patient (III:1)- and healthy control-derived lymphoblast cells demonstrating that RBM10 mRNA from the patient was expressed at similar levels, but with different size. (C) Western Blot performed on patient- and healthy control-derived lymphoblast cells demonstrating that RBM10 protein from the patient was expressed at 70% levels compared with the control, but with different size.

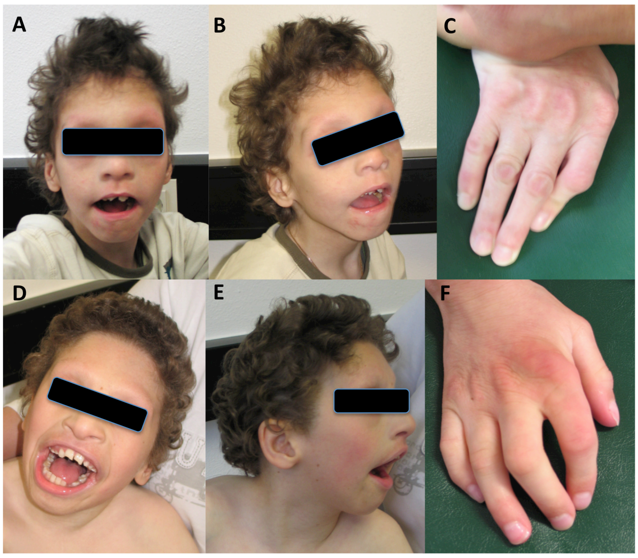

Figure S7. Photos from the two patients reported in this study (Informed consent was obtained from the parents). (A,B,C) photos from patient III:1, (D,E,F) photos from patient III:2.

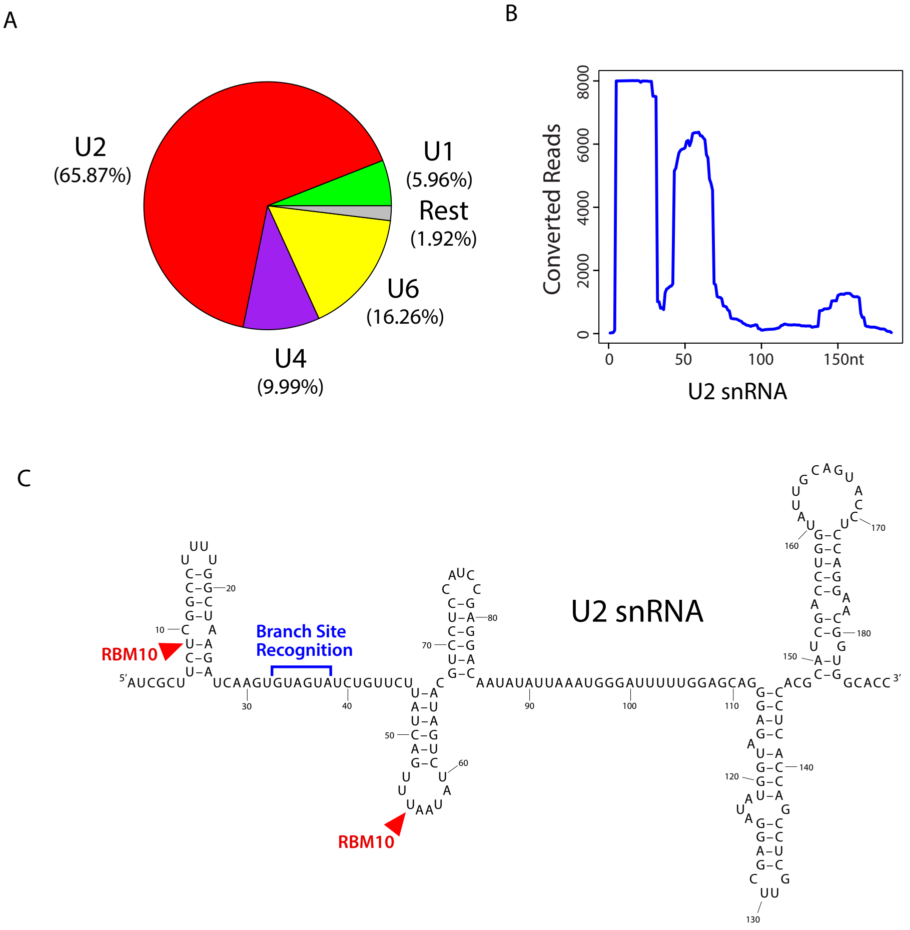

Figure S8. RBM10 binding to U2 snRNA. (A) Distribution of RBM10 PAR-CLIP reads at both major and minor spliceosomal snRNAs. More than 65% of PAR-CLIP reads derived from snRNAs could be mapped to U2. (B) Distribution of RBM10 PAR-CLIP reads containing T-C conversions along the U2 consensus sequence. (C) Two strong crosslinking sites marked with red arrows were close to U2 conserved sequences responsible for branching site pairing.

Table S1. Summary of PAR-CLIP sequencing results. Numbers of (1) sequencing reads, (2) reads uniquely mapped to the human genome (hg19), and (3) uniquely mapped reads containing a T to C conversion. The given percent values are relative to the preceding analysis step. The table also shows the number of clusters and consensus clusters which are present in both replicates.

	Raw Reads	Uniquely Mapped		Converted		Clusters	Consensus Clusters
**Rep 1**	28,038,313	10,746,757	(38.33%)	7,729,493	(71.92%)	240,712	87,957

**Rep 2**	30,408,456	9,895,582	(32.54%)	6,774,681	(68.46%)	218,281

Table S2. Summary of RNA sequencing results.

	Dataset		Total Reads	Mappable	
**HEK-293**	**OE Repl. 1**	**OE**	69,338,829	65,609,940	(94.62%)
		**Control**	95,536,505	89,861,252	(94.06%)
	**OE Repl. 2**	**OE**	159,351,434	150,789,839	(94.63%)
		**Control**	168,587,988	159,667,423	(94.71%)
	**KD 24h**	**KD**	185,800,290	174,812,067	(94.09%)
		**Control**	159,177,660	153,717,538	(96.57%)
	**KD 48h Repl. 1**	**KD**	64,789,423	61,310,079	(94.63%)
		**Control**	67,040,701	63,494,965	(94.71%)
	**KD 48h Repl. 2**	**KD**	156,124,265	146,683,032	(93.95%)
		**Control**	179,362,952	165,152,030	(92.08%)
	**KD 72h**	**KD**	67,078,047	63,515,498	(94.69%)
		**Control**	61,898,132	58,634,043	(94.73%)
**Lymphoblastoid cells**	**Patient**		42,255,602	32,646,743	(77.26%)
	**Normal Repl. 1**		50,015,249	38,752,321	(77.48%)
	**Normal Repl. 2**		33,713,787	24,828,196	(73.64%)

Table S5: Primer sequences

qRT-PCR validation of splicing changes		
**Targets**	**Forward**	**Reverse**
**PCBP2-C**	5’-CGCCAAAATCAATGAGATCC-3’	5’-CCAGTGATGGTAACCTGCCTA-3’
**PCBP2-I1**	5’-TCAGTGGCATTGAATCCAGC-3’	5’-TTATGCAGCCAATCAAATCG-3’
**PCBP2-E**	5'-ACACCGGATTCAGTGCAGGT-3'	5’-CGATTATGCAGCCAATCAAAT-3’
**SAT1-C**	5’-TGAGGAACCACCTCCTCCTA-3’	5’-TTGATCAGCCGCAGTATGTC-3’
**SAT1-I1A**	5’-CGGAAGGTTACAGTCTCTAGC-3’	5’-GCAAAACCAACAATGCTGTG-3’
**SAT1-I1B**	5’-CTGGACTCCGGAAGGTTACA-3’	5’-TGCTGTGTCCTCATTTATCATG-3’
**SAT1-E**	5’-CACTGGACTCCGGAAGGACA-3’	5’-GATCCTATGCCAAAGCCTCT-3’
**POLDIP3-C**	5’-TCGAATCAAAGGGAAAGTGC-3’	5’-AGGGGAACTCCTCTTCAAGC-3’
**POLDIP3-I1**	5’-CCATCCAGGTTCCACAGCAG-3’	5’-GTTTGGCCTGGTGGTTATTG-3’
**POLDIP3-E**	5’-CACCAAAACCATCCAGAATTTA-3’	5’-CCTGAGGCTGCAAACTTCAT-3’
**CASP8-C**	5’-TGGAGAAGAGGGTCATCCTG-3’	5’-TCTTGTTGATTTGGGCACAGA-3’
**CASP8-I1**	5’-CAGCAAAGAGAGAAGCAGCAG-3’	5’-TGGAGAGTCCGAGATTGTCAT-3’
**CASP8-E**	5’-TGGAGAAGAGGGTCATCCTG-3’	5’-CACACAACTCCTCCCCTTTG-3’
**NCOR2-C**	5'-CGCTCAAGGCAGAGAAGAAG -3'	5'-GGTAGCACTGGAGTCGCTGT -3'
**NCOR2-E**	5'- GTGGAGGATGAGGAGATGGA -3'	5'- GTTGTTGACAGTGGCTTCAG -3'
**LEF1-C**	5'-TGGCAGCCCTATTTCAGTTT-3'	5'-TGCAAACCAGTCTGCTGAAC-3'
**LEF1-I1**	5'-CAGGAATCTGCATCAGGTACA -3'	5'-GGAATGAGCTTCGTTTTCCA-3'
**LEF1-E**	5'- CAGGAATCTGCATCAGGTGG -3'	5'- ACAGTCTGGGTTTTCAACAAG -3'
**CREBBP-C**	5'-AACGTCCAGTTGCCACAAG-3'	5'-ACTGAGCCCATGCTGTTCAT-3'
**CREBBP-I1**	5'-CATGCAAGTTTCTCAAGGGAT-3'	5'-TGGACAGAGTGGTTCATTGG-3'
**CREBBP-E**	5'-CCTGTGAGACCTCCAAGGAT-3'	5'-AGCCCATGCTGTTCATCTG-3'
**RBM5-C**	5'-TTGGTGATTCAAGGAAAGCAC-3'	5'-CAAAGCCAATCTTCAAACTTAGG-3'
**RBM5-I1A**	5'-GGATGGAAGCCAATCAGAAA-3'	5'- CAAACTTAGGTCTGGGATTGC-3'
**RBM5-I1B**	5'-CGCCTTCGTGGAGTTTTATC-3'	5'- CCTTGAATCACCAACTTTTTCTG-3'
**RBM5-E**	5'-GCTGATGAAGAGGAAAACAGAAA-3'	5'-CAAACTTAGGTCTGGGATTGC-3'
**CLK2-C**	5'-CGGGGAGATGCCTACTATGA-3'	5'-GCTGCGGTAACTGCTGTTC-3'
**CLK2-I1**	5'-CCGCTCATCTTCGCACAG-3'	5'-GTCCCCTCTCCTAAGGTGCT-3'
**CLK2-E**	5'-AATATCAGCGGGAGAACAGC-3'	5'-GGTGCTAACGATTTCATCGAAG-3'
**ATG16L1-C**	5'-CCCCAGGACAATGTGGATAC -3'	5'-CACACAAGGCAGTAGCTGGT-3'
**ATG16L1-I1**	5'-CAGAGCAGCCACTAAGCGAC-3'	5'-GGGACTGGGAAGGAAGAGAC-3'
**ATG16L1-E**	5'-AGCAGAGCAGCCACGAGAC-3'	5'-CACACAAGGCAGTAGCTGGT-3'
**HOTAIR-C**	5'-CGCAGTGGAATGGAACGGA-3'	5'-AACTCTGGGCTCCCTCTCTC-3'
**HOTAIR-I1**	5'-TGCTCTCAATCAGAAAGGTCC -3'	5'-AACTCTGGGCTCCCTCTCTC-3'
**HOTAIR-E**	5'-ACTGCTCCGTGGGGTCCT-3'	5'-AACTCTGGGCTCCCTCTCTC-3'
**NPNT-C**	5'-TGTCGTTATGGTGGGAGGAT-3'	5'-TGACACTGTCCCCAAGACTG-3'
**NPNT-I1**	5'-GGGACAGTGTCAGCCTTTCT-3'	5'-GTGGTTGGCACACAGCTTTG-3'
**NPNT-E**	5'-TGGCCTATGTCGTTATGGTG-3'	5'-TGGTTGGCACACAGGCTG-3'
**PHACTR2-C**	5'-TGCCTCAGACACTCCAGTTG-3'	5'-GTTCTGTTCGTCGGCTCTTC-3'
**PHACTR2-E**	5'-GGCAGAAGATAAGAAAGCTGG-3'	5'-TGAGGTGGATGAAGTGGATG-3'

**BCOR-C**	5’-GCCGACTGGGAAAGGTTGAA-3’	5’-GTTCTGCAATGGCCTCCTCC-3’
**BCOR-I1**	5’-CCGCTGCTTACTGTGAGCGT-3’	5’-AGTCTTTGGTTGCTGGGTGG-3’
**BCOR-E**	5’-CAACGGGCATTGCAGCGT-3’	5’-AGTCTTTGGTTGCTGGGTGG-3’
**SPTAN1_C**	5’-GGAACTGGGTGAGAAGCGTA-3’	5’-CTCCGACCTCCTCACTTGTC-3’
**SPTAN1_I1**	5’-GGAGCAGATTGACAATCAGAC-3’	5’-CCAACATGCCTTTACGCTTC-3’
**SPTAN1_E**	5'- AGGAGCAGATTGACAATCAATA -3'	5’-TCGCTTCACGGAACAACATA-3’
**UBN1_C**	5’-GGCTTCACCCTACAAATCCA-3’	5’-TGGACAGGAATAGGGACAGG-3’
**UBN1_I1**	5’- CCTGTCCATGTGCTCTCCTT-3’	5’-AGTGCAAGCCAGCCAGAAG-3’
**UBN1_E**	5’-CCTGTCCATGTGCTCTCCTT-3’	5’-GTGAAGCTGAGAAGCACTGT-3’
**PUF60_C**	5’-GCGTCTACGTGGGCTCTATC-3’	5’-CATGTCGATGCTCTTGATGG-3’
**PUF60_I1**	5’- TCTCACCTTTGCAATCGATG-3’	5’- TCCTCCCCCAGCTCATAGTA-3’
**PUF60_E**	5’- CATGGAGCAGAGCATCAAGA-3’	5’-CCGCTGAGCCGCCATCTG-3’
**RBM10_CDS**	5’-CTCTACTATGACCCCAACTCCCA-3’	5’- GTCCGCCTCTCCCCATCCCA-3’
**GAPDH**	5’-TGCACCACCAACTGCTTAGC-3’	5’-GGCATGGACTGTGGTCATGAG-3’

RT-PCR validation of splicing changes

**Targets**	**Forward**	**Reverse**
**UBN1**	5'-AAAGGAGCGAGTGGGACTG-3'	5'-TCACAATTTCCGTGGTACAGCAGGC-3'
**PUF60**	5’-GCCAAGAAGTACGCCATGGA-3’	5'-CTTGTGCTTCATGGTGACGGA-3'
**POLDIP3**	5’-CTTAATGCCAGACCGGGAGT-3’	5'-GTGGTGGAGAAAGCCGCCTGAG-3'
**PCBP2**	5’-TTGACCAAGCTGCACCAG-3’	5'-ATCGTTTGGAATGGTGAGTTC-3'
**CREBBP**	5’-CGGAGGTCGCGTTTACATAA-3’	5'-TGGCACTGAGCCCATGCTGT-3'
**RBM5**	5'-ATTCGAGAAATGATGGAGTC-3'	5'-CTTGTTACAAAGCCAATCTTCAAACT-3'
**SAT1**	5'-CTGCTAGAAGATGGTTTTGGA-3'	5'-CACTCATCACGAAGAAGTCCTCAAG-3'
**SPTAN1**	5’-GATTGGTGGAAAGTGGAAGTG-3’	5'-CTTCTGGAAGTCATCAAACTTCT-3'
**SBF1**	5’-AGCCTGGAGCAGGAGAAGTA-3’	5'-CTTGAGCTGGGCTTTGTCCCCA-3'

Validation of genomic deletion within RBM10

**Name**	**Forward**	**Reverse**
**Within deletion**	5’-AGGTGGTCAGGAGCCGTAG-3’	5’-GTGTTTGGGCCTGTGTGAG-3’
**Flanking deletion**	5’-AGCCCACTTGTCAGAAAACG-3’	5’-ACTGCCAATGTTGTCACTGC-3’

Overlapping PCR for constructing RBM10-PUF fusion protein

**Name**	**Forward**	**Reverse**
**RBM10**	5’-CACCATGGAGTATGAAAGACGTGGTGGTCG-3’	5'-GTAAATTGGGGTACCGGTTGTTTCGAAAATCTTCCAAAAGCTGGGCCTCGTTGAAGCGGGTC-3'
**PUF domain**	5'-CTGCACAAGACAATGGTGACCCGCTTCAACGAGGCCCAGCTTTTGGAAGATTTTCGAAACAAC-3'	5'-TTACTTCTCCAGCTTGGCCAG-3'

Primers targeting the two constitutive GFP exons in pZW2C

**Name**	**Forward**	**Reverse**
**GFP**	5'-AGTGCTTCAGCCGCTACCC-3'	5'-GTTGTACTCCAGCTTGTGCC-3'

Table S6 Summary of phenotypic spectrum in TARP patients and our patients

	Patient 1 (this report)	Patient 2 (this report)	Patient 1 (Gorlin et al., 1970)	Patient 1 (Johnston et al., 2010)	Patient 2 (Johnston et al., 2010)	Patient 3 (Johnston et al., 2010)	Patient 1 (Gripp et al., 2011)
Sex	m	m	m	m*	m*	m*	m
Death at	14 years	alive with 14 y	3 months	5 minutes	8 days	unknown	alive with 3 7/12 y
**Pregnancy/birth**							
normal birth measurements	+	-, low	n.r.	+	-	n.r.	+
oligohydramnios	-	-	n.r.	+	-	n.r.	+
**Neurological signs**							
Severe ID	+	+	n.r.	/	/	/	+
Seizures	+	-	n.r.	-	+	n.r.	
**Body measurements**	at 11 years	at 10 years	3 months				
Short stature	+	+	+	/	/	/	-
Microcephaly	+	+	n.r.				-
**Craniofacial anomalies**							
trigonocephaly	+	+	n.r.	n.r.	n.r.	n.r.	n.r.
low-set ears	+	+	+	+	n.r.	n.r.	+
absence of lateral eyebrows	+	+	-	n.r.	n.r.	n.r.	n.r.
large mouth	+	+	+	n.r.	n.r.	n.r.	n.r.
thin upper lip	+	+	+	n.r.	n.r.	n.r.	n.r.
micrognathia	+	+	+	+	+	+	+
glossoptosis	-	-	-	n.r.	+	+	n.r.
hypoplastic uvula	+	+	n.r.	n.r.	n.r.	n.r.	n.r.
cleft palate	+	+	+	+	+	+	+
**Anomalies of limbs**							
broad hands	+	+	n.r.	+	n.r.	n.r.	-
ulnar deviation of hands	+	+	n.r.	+	n.r.	n.r.	-
talipes equinovarus	+	+	+	+	n.r.	+	-
rocker bottom feet	-	-	-	+	n.r.	n.r.	-
**Internal malformations**							
Dandy-Walker malformation	+	-	-	n.r.	n.r.	n.r.	-
Cerebellar vermis hypoplasia	-	+	-	n.r.	n.r.	n.r.	+

heterotopia	-	-	-	n.r.	n.r.	n.r.	+
Optic atrophy	+	-	-	n.r.	n.r.	n.r.	+
cataracts			+				
Diaphragmatic hernia	+	-	-	n.r.	n.r.	n.r.	-
Atrial septal defect	-	+	+	+	n.r.	(+)	+
Ventricular septal defect	+	-	-	-	-	-	-
persistence of left sup.v. cava	n.r.	n.r.	+	n.r.	n.r.	n.r.	+
hypoplasia of lung	n.r.	n.r.	n.r.	+	n.r.	n.r.	+
micropenis	+	-	n.r.	n.r.	n.r.	n.r.	n.r.
undescended testis	+	+	+	unilateral	n.r.	n.r.	n.r.
Others:							
Hearing loss	+	-	n.r.	n.r.	n.r.	n.r.	+
Renal insufficieny	+	-	n.r.	n.r.	n.r.	n.r.	+
Myelodysplastic syndrome	+	-	n.r.	-	-	-	n.r.
Colitis ulcerosa	+	-	n.r.	-	-	-	n.r.
Scoliosis	-	+	n.r.	n.r.	n.r.	n.r.	n.r.
RBM10 mutation	in frame 1292 nt deletion	in frame 1292 nt deletion	c.1893_1894insA	c.1235G>A	c.1235G>A	c.1235G>A	

*: no photographs published

Supplementary Methods

Cell lines

Stable human embryonic kidney (HEK) 293 T-REx Flp-In cell lines inducibly expressing FLAG/HA-tagged wild type and mutant RBM10 respectively were generated and maintained as described previously (Landthaler et al., 2008) with the following minor modifications. First, the full-length coding sequence (CDS) of RBM10 wildtype (WT) and mutant (MUT) were ligated into pENTR/D-TOPO vector (Invitrogen) respectively according to the manufacturer's protocol, and then exchanged from pENTR/D-TOPO into pFRT_TO_DESTFLAGHA vector (Addgene: 26361) by LR reaction using Gateway LR Clonase II enzyme mix (Invitrogen) to generate the expression plasmids. Second, a serial of doxycycline concentrations: 0, 1, 5, 10, 20, 50, 100, 200, 400, 1000 ng/mL respectively, for induction were tested by western blot to obtain optimal protein expression levels.

PAR-CLIP

The PAR-CLIP method described here is modified from (Hafner et al. 2010; Lebedeva et al. 2011).

(1) 4-thiouridine labeling, doxycycline induction and crosslinking
Stable HEK293 Flp-In T-REx cells inducibly expressing FLAG/HA-tagged RBM10 were grown in selective medium. Typically, five 15-cm plates of 80 % confluent cells were used for one PAR-CLIP experiment. 4-thiouridine (4SU) and doxycycline (Dox) were diluted in selective medium together, added to the cells to the final concentration of 100 μM, 10 ng/mL respectively and incubated for 16 hours. After labeling and induction, the medium was aspirated from the plates and the cells were crosslinked on ice using Stratalinker (Stratagene) with 365nm UV-lamps (Energy setting: 1500 μJ x 100/cm2). Cells were scraped off in cold PBS and pelleted by centrifugation at 1,000 x g for 5 min at 4°C.

(2) Cell lysis and immunoprecipitation (IP)

Crosslinked Cell pellet was lysed in 3 volumes of high salt lysis buffer (50 mM Tris-HCl pH 7.2, 500 mM NaCl, 1% NP40, 1 mM DTT, complete protease inhibitor (Roche)) and sonicated with ultrasonic homogenizer Sonopuls HD 2070 (BANDELIN) (settings: 45 SEC, CYCLE 2 x 10%, POWER 70 %). Cell lysate was cleared by centrifugation at 13,000 rpm for 15 min at 4ºC using Sorvall RC-6 Plus Superspeed Centrifuge (Thermo Scientific) and filtered through a 5 μm membrane syringe filter (Pall). The filtered lysate was partially digested with RNaseT1 (Fermentas) at a final concentration 1U/μl for 15 min in a room temperature water bath and subsequently cooled on ice for 5 min. RBM10-RNA complexes were immunoprecipitated from partially digested cell lysate using monoclonal anti-FLAG (Sigma, F1804) conjugated to magnetic Protein G Dynabeads (Invitrogen) and incubation for 1 hour at 4ºC on the rotation wheel. For 1 ml of cell lysate, 25 μl beads and 10 μg of antibody were used. Beads were washed three times with IP wash buffer (50 mM Tris-HCl pH 7.5, 300 mM KCl, 0.5% NP-40, 0.5 mM DTT, protease inhibitor cocktail) and resuspeneded in original bead volume of IP wash buffer, subsequently treated wtih RNase T1 at final concentration of 10 U/μl for 5 min in room temperature water bath. The RNase T1 treated beads were immediately washed three times with ice-cold high salt wash buffer (50 mM Tris-HCl pH 7.5, 500 mM KCl, 0.05% NP-40, 0.5 mM DTT, protease inhibitor cocktail), three times with 1x NEB buffer 3 and resuspended in one bead volume of NEB buffer 3 containing 0.5 U/μl Calf Intestinal Phosphatase (NEB). Dephosphorylation was performed at 37ºC in a thermomixer (Eppendorf) with shaking at 800 rpm for 30 min. Beads were then washed twice with phosphotase wash buffer (50 mM Tris-HCl pH 7.5, 20 mM EGTA, 0.5% NP-40), twice with PNK buffer without DTT (50 mM Tris-HCl pH 7.5, 50 mM NaCl, 10 mM MgCl2) and then labeled with 5 μl γ-32P-ATP (final concentration of 0.5μCi/μl) (Perkin-Elmer, NEG 502A) in PNK buffer with 5 mM DTT by T4 polynucleotide kinase (T4 PNK) (NEB). The labeling reaction was carried out at 37ºC with 800 rpm shaking for 30 min. ATP (Fermentas) was added to a final concentration of 100 μM and the reaction was further incubated for 5 min. After radioactive labeling, the beads were washed five times with 800 μl PNK buffer without DTT and resuspended in 40 μl of 2x SDS-PAGE loading buffer (20% glycerol (v/v), 160 mM Tris-HCl pH 6.8, 4% SDS (w/v), 200 mM DTT, 0.2% bromophenol blue).

(3) SDS-PAGE and electroelution of RNA

Beads in SDS loading buffer were boiled at 95ºC for 5 min and placed on magnetic rank. The supernatant was loaded into two wells of an SDS gel (NuPAGE Novex 4-20% BT Gel, Invitrogen). The gel was run at 200 V for ~1 hour in 1 x MOPS SDS running buffer (Invitrogen), exposed for 10 min on a phosphorimaging screen and visualized on Fujifilm FLA-7000 PhosphorImager (Fujifilm). The radioactive band corresponding to RBM10-RNA complexes (~130 KDa) was cut out from the gel and placed into D-Tube Dyalyzer Kit MWCO 3.5kDa (Novagen). 800 μl 1 x MOPS SDS running buffer was filled into the tube and RBM10-RNA complexes were electroeluted from the gel for 1.5 h at 100V and 2 min by reversing the current in 1 x MOPS SDS running buffer. The elution containing RBM10-RNA complexes (~700 μl) was taken out into nuclease free low binding tubes (Eppendorf). Subsequently, RBM10 was digested by adding equal volume of 2 x proteinase K buffer (200 mM Tris-HCl pH 7.5, 150 mM NaCl, 12.5 mM EDTA, 2% SDS) with proteinase K (Roche) at final concentration of 2 mg/ml and incubation at 55ºC for 60 min. RNA was then recovered by phenol-chloroform extraction and ethanol precipitation with GlycoBlue (Ambion).

(4) RNA cloning and sequencing

Sequencing libraries were constructed using the small RNA cloning protocol (Hafner et al. 2008). The RNA pellet was resuspended in ligation mix containing: 2 μl of 10× RNA ligase buffer without ATP, 10 μl of PEG8000 (50%), 1 μl of 100 μM preadenylated 3'adapter, 6 μl H2O. The mixture was denatured at 95ºC for 30 sec and put on ice for 5 min. 1 μl T4 RNA Ligase K227Q (NEB, M0351S) was added and the reaction was incubated at 16 ºC for 16 hours. The radiolabeled 19 and 35 nt RNA markers were included as control. Ligation product was mixed with 20 μl 2 x formamide RNA loading dye (50 mM EDTA-NaOH pH 8.0, 0.05 % (w/v) Bromophenol blue in formamide), separated by 15% denatured PAGE (UreaGel - SequaGel - System, National Diagnostics, EC-833) and exposed for at least 2 hours. The 3'adaptor ligated product and size markers were cut out from the gel and eluted by 0.4 M NaCl for 16 hours at 4ºC with 800 rpm shaking in thermomixer, respectively. The eluted RNA was precipitated by ethanol, using Glycoblue. The pellet was resuspended in 5'ligation mix containing: 2 μl of 10× T4 RNA Ligase buffer with ATP, 10 μl PEG8000 (50%), 5 μl H2O and 1 μl of 100 μM 5' ligation

adapter and denatured at 95ºC for 30 sec. 2 µl T4 RNA Ligase 1 (NEB, M0204) was added and the reaction was incubated for 1.5 hours at 37ºC. The ligation product including size markers were separated by 12% denatured PAGE and exposed overnight. The 5'adaptor ligated product and size markers were cut out from the gel and eluted by 0.3 M NaCl for 16 hours at 4ºC with 800 rpm shaking in thermomixer. The eluted RNA was precipitated by ethanol with 1 µl of 100 µM reverse transcription (RT) primer, using Glycoblue. The RNA pellet was resuspended in 14.3 µl RT mix containing: 5.6 µl H2O, 1.5 µl of 0.1 M DTT, 4.2 µl 10× dNTPs (2 mM each), 3 µl 5× First Strand buffer and denatured at 95ºC for 30 sec and put on ice. The mixture was incubated at 50ºC for 3 min, then 0.75 µl of Superscript III (invitrogen) was added and incubated at 42ºC for 30 min. After RT, 85 µl H2O was added to the reaction. The diluted reverse transcription (RT) product was amplified by Phusion polymerase in the following 50 µl reaction system: 27.75 µl H2O, 10 µl 5x Phusion HF buffer, 1.25 µl dNTPs (10 mM), 0.25 µl 5' PCR primer (100 µM), 0.25 µl 3' PCR primer (100 µM), 0.5 µl Phusion DNA Polymerase, 10 µl RT product. The optimal PCR cycle was determined by assessing aliquots of PCR products taken out from different cycles on 2.5% low melting agarose gel (Lonza). Three PCR reactions were setup with optimal cycle, precipitated by ethanol with glycoblue, re-dissolved in nuclease free H2O, and separated by 2.5% low melting agarose gel (Lonza). The band within correct size range was cut out, extracted by Qiagen gel extraction kit and resuspended in 15 µl H2O. The purified library was quantified by Qubit, the size range was assessed by Bioanalyzer DNA 1000 chip, sequenced on an Illumina HiSeq for 1 x 50 cycles following the manufacturer's protocol.

Western blot

Cells were lysed in RIPA lysis buffer (50 mM Tris pH 8.0, 150 mM NaCl, 1.0% NP-40, 0.5% sodium deoxycholate, 0.1% SDS, complete protease inhibitor (Roche)) and sonicated by using Biorupter (UCD-300, Diagenode) with the setting: high energy mode, 10 cycles, 30 sec-on and 30 sec-off each. Additional cycles were performed if the cell lysate was viscous after sonication. The cell lysate was centrifuged at 18,000 x g for 10 min at 4°C. The supernatant in 1 x SDS-PAGE loading buffer was boiled at 95°C for 5 minutes, centrifuged at 18,000 x g for 2 min and resolved by 10% SDS-

PAGE in 1 x SDS running buffer (25 mM Tris base,190 mM glycine, 0.1% SDS) at 160 V for ~1 hour until the dye runs to the bottom of the gel chamber. Proteins were transferred to PVDF membrane (GE healthcare) using semi-dry blotting apparatus (BioRad) at 20 V for ~1 hour for RBM10. The membrane was blocked in 5% non-fat milk, washed once with 1 x TBST (50 mM Tris.HCl, pH 7.4, 150 mM NaCl, 0.1% Tween 20) and incubated with primary antibody diluted in 1 x TBST for 1 hour at room temperature or 4°C overnight with gentle shaking. After primary antibody incubation, the membrane was washed three times with 1 x TBST, 5 min each, and incubated with HRP-conjugated secondary antibody at room temperature for 1 hour. The secondary antibody incubated membrane was washed three times with 1 x TBST, 5 min each, developed with SuperSignal Kit (Thermo) and detected by LAS-4000 imaging system (Fujifilm) following the manufacturer's instructions. The following antibodies were used: rabbit polyclonal anti-RBM10 (Abcam, ab26046, 1:2000), mouse monoclonal anti-HA (Covance, MMS-101P, 1:4000), monoclonal anti-FLAG (Sigma, F1804, 1:4000), and secondary HRP-conjugated goat anti-mouse or human IgG (Santa Cruz, 1:2000).

Prediction of subcellular localization of RBM10 wild type and mutant

The amino acid sequences of RBM10 wild type and mutant proteins were submitted to the software PSORTII (http://psort.hgc.jp) for the localization prediction and signal analyses. The 930aa-long RBM10 was predicted with high confidence to reside in the cell nucleus mainly due to the existence of 2 nuclear localization signal (NLS) close to both N-terminal (a weaker signal) and to C-terminus (a stronger signal). The later classic NLS was however deleted in the patient RBM10 sequences, and thus largely reduced its localization probability in the nucleus.

Immunofluorescence

The cells were induced with appropriate concentration of doxcycline for 16 hours when induction of the protein expression is needed. Cells grown on sterile cover slides were gently washed once with 1 x PBS, fixed with freshly prepared 3.7% paraformaldehyde (PFA) (Invitrogen) in 1 x PBS for 20 min at room temperature, and

rinsed for 2 min in 1 x PBS. The fixed cells were permeabilized in 0.1% Triton X-100 in 1 x PBS for 15 min at room temperature, washed 3 times with 1 x PBS, and blocked by 5.0% BSA in PBS for 1 hour at room temperature. The blocked cells with the excess blocking solution drained away were transferred to humid chamber, incubated with 200 μl primary antibody freshly diluted at in antibody dilution solution (0.5% BSA in PBS) for 2 hours at room temperature or 4 °C overnight. Subsequently, the cells were washed 3 times with 1 x PBS, 5 min each, and blocked for 20 min at room temperature. After draining away the blocking solution, the cells were incubated with 200 μl fluorochrome-conjugated secondary antibody freshly diluted in antibody dilution solution for 40 min at room temperature. After draining away the excess secondary antibody solution, the cells were stained with 1:5000 DAPI (Invitrogen) diluted in antibody dilution solution for 5 min at room temperature, washed 3 times with 1 x PBS, 5 min each, and air dried. The dried cells were then mounted on the microscopic glass slide with Prolong Gold Antifade Reagent (Invitrogen) and observed under Leica SP5 confocal microscope (Leica) following the manufacturer's manual. The following antibodies were used: rabbit polyclonal anti-RBM10 (Abcam, ab26046, 1:200), rabbit polyclonal anti-RBM10 (NB100-55265, 1:200), mouse monoclonal anti-HA (Covance, MMS-101P, 1:400), Alexa Fluor 488 goat anti-rabbit IgG (Invitrogen, A-11034, 1:400), and Alexa Fluor 568 goat anti-mouse IgG (A-11004, 1:600).

EMSA

FLAG/HA tagged RBM10 was purified from five 15-cm plates established HEK293 stable cell lines following the PAR-CLIP IP procedure. The cells were induced with 1 mg/mL doxycycline for 16 hours. After 3 times washing with IP wash buffer, the protein on the beads was eluted twice with 100 μl 3XFLAG peptide (Sigama, T6664) at final concentration 250 ng/μl in 1 x TBS (50 mM Tris.HCl, pH 7.4, 150 mM NaCl) for 30 min at 4ºC with rotation each. The purified protein was assessed by Coomassie blue staining and western blot using mouse monoclonal anti-FLAG (Sigma, F1804, 1:4000), and quantified by Nanodrop.

RNA oligos were synthesized from Integrated DNA Technologies, diluted in nuclease free H_2O. 10 μM RNA oligo was radioactively labeled with γ-32P-ATP (Perkin-Elmer, NEG 502A) by T4 polynucleotide kinase (T4 PNK) (NEB) following the

manufacturer's protocol, and purified by G-25 columns (GE healthcare) following the manufacturer's manual. 100 pM radiolabeled RNA was incubated with different amounts of purified RBM10 in a 20 µ reaction system at room temperature for at least 2 hours in 1X binding buffer (10 mM Tris-HCl, pH 8.0, 25 mM NaCl, 0.1 mM EDTA, 0.1 mg/mL of tRNA, 5 µg/mL of heparin, 0.01% IGEPAL CA630 (Sigma)). The reaction was loaded on a 6 % native PAGE and run in cold at 200 V for 20 min. The gel was exposed overnight and visualized at Fujifilm FLA-7000 PhosphorImager (Fujifilm).
